# Quantum Fisher information in a strange metal

**DOI:** 10.1038/s41567-026-03298-0

**Published:** 2026-06-15

**Authors:** Federico Mazza, Sounak Biswas, Xinlin Yan, Andrey Prokofiev, Paul Steffens, Qimiao Si, Fakher F. Assaad, Silke Paschen

**Affiliations:** 1 Institute of Solid State Physics, TU Wien, Vienna, Austria; 2 Institut für Theoretische Physik und Astrophysik, Universität Würzburg, Würzburg, Germany; 3 Institut Laue-Langevin, Grenoble, France; 4 Department of Physics and Astronomy, Extreme Quantum Materials Alliance, Rice Laboratory for Emergent Magnetic Materials, Smalley-Curl Institute, Rice University, Houston, TX USA; 5 Würzburg-Dresden Cluster of Excellence ctd.qmat, Würzburg, Germany

**Keywords:** Phase transitions and critical phenomena, Theoretical physics

## Abstract

A strange metal is an exotic state of correlated quantum matter, and intensive efforts are ongoing to understand its nature. Here we show that the quantum Fisher information—a concept from quantum metrology—may provide helpful insights. We use inelastic neutron scattering and quantum Monte Carlo simulations to study quantum critical fluctuations of the Kondo destruction type, which are considered to underlie strange metal behaviour in heavy-fermion compounds. We find that the associated quantum Fisher information increases strongly and without a characteristic scale as the strange metal forms with decreasing temperature. This provides evidence for a quantum state with high multipartite entanglement and offers a positive descriptor of strange metallicity that points towards its microscopic basis. Our work opens a direction for future studies across a range of strange metal platforms.

## Abstract (web summary)

A strange metal is a strongly correlated quantum state that occurs in many different materials. Now, quantum Fisher information is shown to increase as the strange metal forms, indicating that the state has high multipartite entanglement.

## Main

Strange metal behaviour refers to a linear temperature dependence of the electrical resistivity at low temperatures instead of the square-in-temperature Fermi liquid form. First recognized in cuprate high-temperature superconductors, strange metals are being identified in an increasing number of materials classes, from heavy-fermion, pnictide and organic compounds to frustrated-hopping and moiré flat-band systems^1^. Heavy-fermion compounds have played an important role in the search for other salient features of strange metallicity^2^, and a Fermi volume jump^3^, dynamical scaling in the spin response^4^ and charge (or current) response^5^, and the suppression of shot noise^6^ have been evidenced. All of them are consistent with the static Kondo screening transitioning, in the zero-temperature limit, from being in place to being absent^7–9^, a scenario that is actively pursued with various theoretical techniques^10–13^. As evidence for these features is accumulating in other strange metal platforms^1^ and theoretical efforts are made^14–17^ to understand these systems in Kondo-based frameworks, it is possible that the Kondo destruction (or breakdown) scenario is pertinent beyond the heavy-fermion setting. However, very different scenarios are also considered^18–20^ and a unified understanding is still lacking.

Here we explore the potential of a quantum information-inspired probe—the quantum Fisher information (QFI)—to make progress. As recently shown theoretically^21^, the QFI can be defined for condensed-matter systems in thermal equilibrium via a Kubo response function

1 $${f}_{{\rm{Q}}}(T)=\frac{4}{\pi }{\int }_{0}^{\infty }\tanh \left(\frac{\hslash \omega }{2{k}_{{\rm{B}}}T}\right){\chi }^{{\prime\prime} }(\omega ,T){\rm{d}}(\hslash \omega )$$

involving the imaginary part of a dynamical susceptibility *χ*″(*ω*, *T*), for instance, the dynamical spin susceptibility that can be derived from inelastic neutron scattering (INS) experiments. In this formulation, called the QFI density^22^, the susceptibility is an intensive quantity, that is, it is counted per site or moment. The benefit of this tool is that it extracts the entanglement content of the quantum correlations contained in *χ*″(*ω*, *T*) and, thus, provides complementary information to dynamical scaling analyses. A prediction of direct pertinence for the present work is that at strongly entangled quantum phase transitions, the QFI is expected to diverge in the *T* = 0 limit, whereas no signature should appear at a thermal phase transition^21^. By contrast, in a spin-chain material, enhanced values of the QFI were found to be tied to the Néel order parameter and to decrease as the order is suppressed^22^. Motivated by the finding that at the Kondo destruction quantum critical point (QCP) of a Kondo impurity model, the entanglement entropy becomes long ranged^23^, we set out to study a strange metal heavy-fermion compound by INS experiments. In what follows, we make the case that the QFI due to local fluctuations associated with the Kondo destruction process, observed at a strange metal QCP, increases strongly with decreasing temperature as the strange metal develops, providing evidence for a state with enhanced multipartite entanglement (Supplementary Section B).

**Fig. 1:**
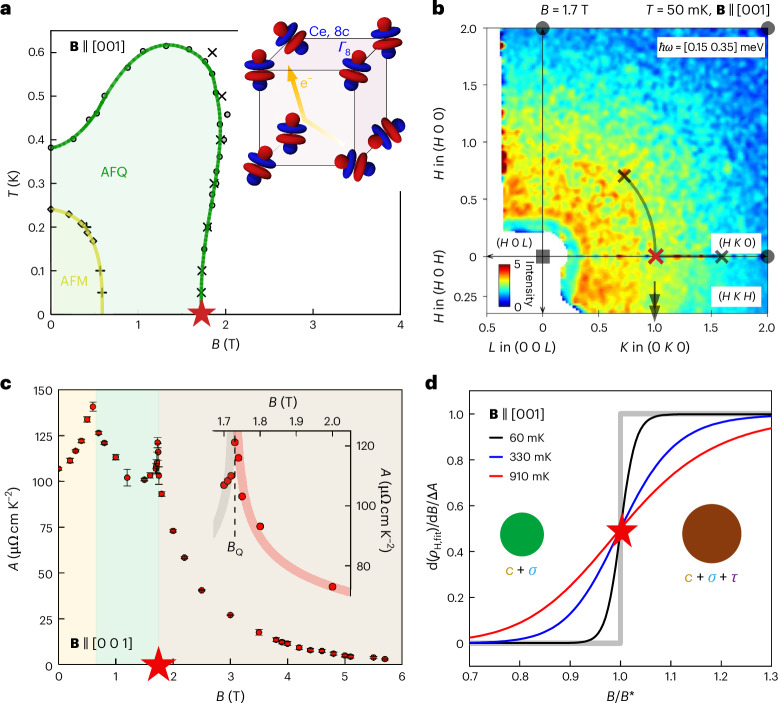
Ce_3_Pd_20_Si_6_ with orbital moments undergoing Kondo destruction. **a**, Temperature–magnetic field phase diagram of Ce_3_Pd_20_Si_6_, for a magnetic field applied along the crystallographic [0 0 1] direction. At the QCP (red star; *B*_Q_ ≈ 1.73 T) studied here, the AFQ order is continuously suppressed^24^. The antiferromagnetic (AFM) order is suppressed already at *B*_M_ ≈ 0.7 T (ref. ^33^). Both QCPs feature signatures of Kondo destruction^24,33^. Inset: sketch of the crystal structure, displaying only the 4f orbitals of the magnetically active Ce atoms at the 8*c* position, which assume a *Γ*_8_ quartet ground state. **b**, Constant-energy map at 50 mK, for the same field configuration as in **a**, obtained by integrating time-of-flight data within the indicated energy range, and within ±0.08 reciprocal lattice units (r.l.u.), that is, wavevectors in units of $\frac{2\pi }{a}$, where *a* is the length of the unit cell in the orthogonal momentum direction^26^. The red cross, grey square and grey circles indicate the position studied with the triple-axis spectrometer ThALES, the direction of the neutron beam and the nuclear Bragg peaks, respectively (note that nuclear Bragg peaks in this structure exist only at all-even and all-odd Miller indices^30^). The double arrow indicates where the magnetic Bragg peak of the AFQ order would appear (at (1 1 1)). The grey-shaded lines from the red cross to the grey crosses indicate trajectories remeasured on ThALES (Supplementary Section C and Supplementary Fig. 2). **c**, Electrical resistivity follows the Fermi liquid form *ρ* = *ρ*_0_ + *AT*^2^ at the lowest temperatures, in shrinking temperature ranges on approaching the critical fields, and with a strongly enhanced *A* coefficient on approaching those fields, consistent with divergences for fields near *B*_Q_ (inset). At both critical fields and in quantum critical fans emerging from them, the resistivity assumes the strange metal form $\rho ={\rho }_{0}^{{\prime} }+{A}^{{\prime} }T$ (ref. ^24^). **d**, Differential Hall resistance jumps in the extrapolated zero-temperature limit^24^, both at *B*_Q_ as shown here and at *B*_M_ (ref. ^33^). This is understood as resulting from the spin degree of freedom *σ* of the *Γ*_8_ quartet being incorporated into the Fermi surface at *B*_M_, and the orbital degree of freedom *τ* being incorporated at *B*_Q_ (refs. ^24,33^), via the Kondo destruction (or construction) mechanism. Panels adapted with permission from: **a**, ref. ^26^, APS; **b**,**c**, ref. ^24^, National Academy of Sciences.

We chose the heavy-fermion metal Ce_3_Pd_20_Si_6_ for this study because quantum criticality of the Kondo destruction type, associated with strange metal behaviour, has been identified in previous experiments^24^ and because large single crystals suitable for INS experiments are available^25^. We focus on the material’s magnetic-field-induced strange metal QCP at 1.73 T (applied along the crystallographic [0 0 1] direction), where a phase with antiferroquadrupolar (AFQ) order is continuously suppressed (Fig. 1a)^26^ and where the quadrupole moments are expected to undergo quantum critical fluctuations of the Kondo destruction type^24^. This means that the fluctuations are between bare quadrupole moments and Kondo-screened ones, a process that is predominantly local in real space and, thus, broad in momentum space. Although neutrons do not directly couple to electric quadrupoles, small secondary magnetic dipole moments can be induced by a magnetic field on top of the primary quadrupole moments, which then act as their magnetic markers^27^ (Supplementary Section E). A hallmark of this effect is the initial increase in the AFQ ordering temperature with field (Fig. 1a), before the phase collapses in the Kondo destruction transition^28^.

As we are interested in multipartite entanglement associated with the critical Kondo destruction process, we have selected the wavevector $(0\,\bar{1}\,0)$ for our study, which is far away from the AFQ ordering wavevector (1 1 1) identified by neutron diffraction via the markers^26,28^. This minimizes contributions from magnetic order parameter fluctuations—fluctuations between mutually aligned and unaligned moments, which are generally not considered a source of strange metallicity^29^ (Supplementary Section A). Furthermore, this choice avoids the contamination of the expected quasielastic quantum critical signal with magnetic Bragg or quasi-Bragg intensity, as well as structural Bragg intensity, as there is also no structural Bragg peak at this position in momentum space^30^. This is vital for data analysis (Supplementary Section C). The very presence of an appreciable intensity detected at this wavevector is remarkable and, by itself, a confirmation of the local nature of the underlying quantum criticality. Note that at fields away from the quantum critical field, the intensity at $(0\,\bar{1}\,0)$ as well as the broad intensity distribution as such are suppressed^26^ (Supplementary Section C and Supplementary Fig. 1).

As shown previously^24^, a magnetic field applied along [0 0 1] drives the material across a two-stage Kondo destruction transition^31,32^. At large fields, both spin (*σ*) and orbital (*τ*) degrees of freedom of the 4*f*^1^ *Γ*_8_ quartet ground state of the magnetically active Ce atoms situated at the 8*c* site (Fig. 1a, inset) are Kondo screened by the conduction electrons (*c*), and the Fermi surface is large (Fig. 1d, brown circle). With a decreasing magnetic field, at *B*_Q_ ≈ 1.73 T, Kondo screening first breaks up for the quadrupole moments, leading to a jump in the Fermi volume to an intermediate size (Fig. 1d, green circle) and AFQ order with the ordering wavevector (1 1 1)^28^. With further decreasing field, at *B*_M_ ≈ 0.7 T, Kondo screening breaks up for the spin degree of freedom^33^, leading to a small Fermi surface that contains only the conduction electrons, and antiferromagnetic (AFM) order with the incommensurate ordering wavevector (0 0 0.8)^28^. Near both critical fields, the effective mass as probed by the *A* coefficient of the Fermi liquid form Δ*ρ* = *AT*^2^ is strongly enhanced (Fig. 1c) before, at the two QCPs, the strange metal linear-in-temperature form prevails down to the lowest temperatures^24^.

**Fig. 2:**
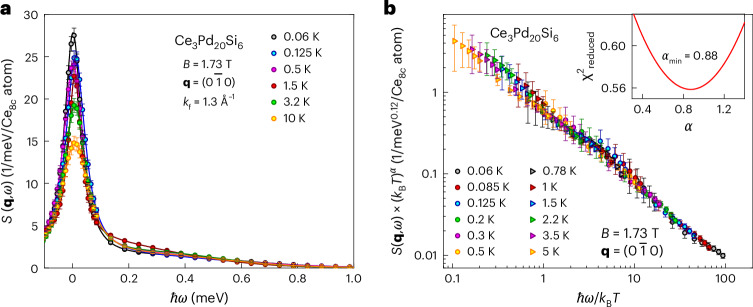
Dynamical spin correlation function and dynamical scaling analysis of Ce_3_Pd_20_Si_6_. **a**, Selected isotherms of the dynamical spin correlation function *S*(**q**, *ω*) versus energy *ℏω*, measured at ${\bf{q}}=(0\,\bar{1}\,0)$ and in a magnetic field of 1.73 T applied along [0 0 1]. **b**, *S*(**q**, *ω*) from **a**, multiplied with ${({k}_{{\rm{B}}}T)}^{\alpha }$ and plotted versus *ℏω*/*k*_B_*T*. Data in the temperature range of 0.06–5 K and for energy transfers in the range of 0.025–0.58 meV show the best overlap for the exponent *α* = 0.88 ± 0.02, as seen from the minimum in the quality factor ${\chi }_{{\rm{reduced}}}^{2}$ of the minimization procedure (inset), a scaling that is compatible with Kondo destruction quantum criticality as described in ref. ^7^. The error bars result from statistical errors and uncertainties in other quantities via error propagation (Supplementary Section D). Source data

We now turn to the INS data of Ce_3_Pd_20_Si_6_ measured down to temperatures of 60 mK. The experiment was performed at the cold-neutron triple-axis spectrometer ThALES (Institut Laue-Langevin), which is the state of the art in terms of the combination of high neutron flux and excellent energy resolution, that is, 0.035 meV (half-width at half-maximum) for the chosen final neutron wavevector *k*_f_. Extreme care was taken to remove all background contributions and to bring the data into absolute units (Supplementary Sections C and D). The thus-obtained dynamical spin correlation function *S*(**q**, *ω*, *T*), measured at ${\bf{q}}=(0\,\bar{1}\,0)$, is shown in Fig. 2a. The extended dynamical mean-field theory of Kondo destruction quantum criticality^7^ predicts the dynamical spin susceptibility *χ*(**q**, *ω*, *T*) at the ordering wavevector **q** = **Q** to exhibit the scaling form

2 $$\chi ({\bf{q}},\omega ,T)=\frac{1}{A{T}^{\alpha }W(\hslash \omega /{k}_{{\rm{B}}}T)}.$$

Here we measure the imaginary part *χ*″(**q**, *ω*, *T*) at ${\bf{q}}=(0\,\bar{1}\,0)$ which, as discussed above, is far away from any magnetic (and structural) Bragg peak. As *χ*″(**q**, *ω*, *T*) is expected to decrease (smoothly) away from **Q** (ref. ^7^), our data represent a lower bound of the quantum critical fluctuation strength (Supplementary Section A). Here *χ*″(**q**, *ω*, *T*) is related to *S*(**q**, *ω*, *T*) via the fluctuation–dissipation theorem^34,35^ as

3 $${\chi }^{{\prime\prime} }({\bf{q}},\omega ,T)=\pi (1-{{\rm{e}}}^{-\hslash \omega /{k}_{{\rm{B}}}T})S({\bf{q}},\omega ,T).$$

A minimization procedure of our *S*(**q**, *ω*, *T*) data for energies below 0.58 meV and temperatures below 5 K produces the best data collapse for the exponent *α* = 0.88 ± 0.02 (Fig. 2b). The excellent quality of the scaling, together with the fractional exponent *α*, provides strong evidence for the beyond-order-parameter nature of quantum criticality. The fact that the scaling form of equation (2) describes our data so well far away from the ordering wavevector of the AFQ phase indicates that contributions from order parameter fluctuations are small in Ce_3_Pd_20_Si_6_, at least on the scale of the energy resolution of the experiment. The broad intensity distribution in the reciprocal-space map (Fig. 1b) supports this assessment (Supplementary Section A).

**Fig. 3:**
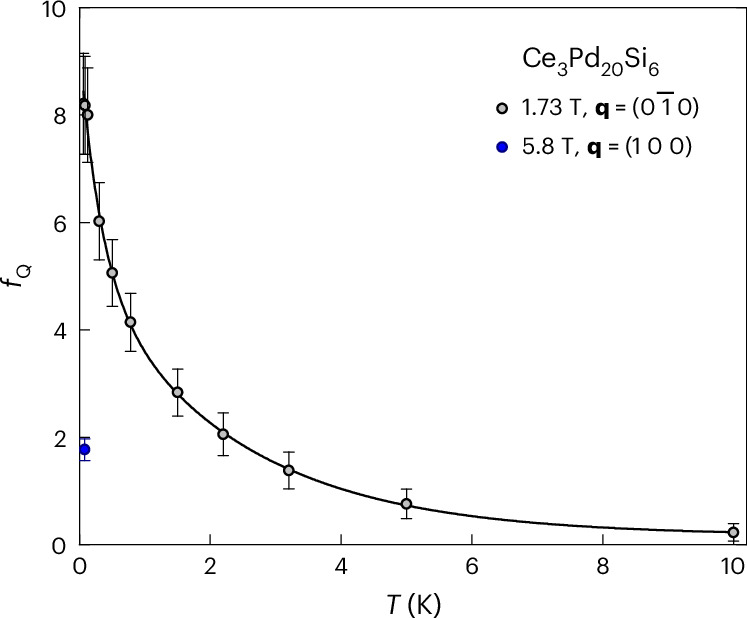
QFI density of Ce_3_Pd_20_Si_6_. The black data points correspond to the ones presented in Fig. 2a, but all the measured isotherms were analysed and the entire accessed energy range from 0 to 1.5 meV was used for the integration, yielding *f*_Q_ = 8.2 ± 0.9 at the lowest temperature of 60 mK. Stopping the integration at 0.58 meV, where the dynamical scaling ceases to be obeyed, yields *f*_Q_ = 7.6 ± 0.8. The blue data point corresponds to a measurement performed at a magnetic field of 5.8 T, far away from the quantum critical field, at 80 mK and with a final neutron wavevector of *k*_f_ = 1.15 Å^−1^ instead of *k*_f_ = 1.3 Å^−1^ used for all other measurements, which increased the instrumental resolution but limited the accessed energy range to 1 meV (Supplementary Section H). The error bars result from statistical errors and uncertainties in other quantities via error propagation (Supplementary Section D). Source data

Next, we determine the temperature-dependent QFI density as

4 $${f}_{{\rm{Q}}}(T)=4{\int }_{0}^{\infty }\tanh \left(\frac{\hslash \omega }{2{k}_{{\rm{B}}}T}\right)(1-{{\rm{e}}}^{-\hslash \omega /{k}_{{\rm{B}}}T})S(\omega ,T){\rm{d}}(\hslash \omega )$$

from the different *S*(**q**, *ω*, *T*) isotherms at ${\bf{q}}=(0\,\bar{1}\,0)$. As our data extend only up to 1.5 meV, we terminate the integral at this energy (Fig. 3 and Supplementary Section I discuss integration range effects). The resulting *f*_Q_ shows a pronounced increase with decreasing temperature, by almost a factor of 40 when cooling from 10 K to 60 mK (Fig. 3), indicating that entanglement is building up as the Kondo destruction QCP is approached. The temperature dependence is smooth, with no sign of a characteristic energy scale or saturation trend. Note that fluctuations from a classical phase transition freeze out below the ordering temperature as spectral weight accumulates near the ordering wavevector and ultimately becomes Bragg intensity. As the tanh filter function in equation (1) suppresses contributions with *ℏω* < 2*k*_B_*T*, *f*_Q_ will then decrease instead of showing a scale-free increase.

At the lowest accessed temperature of 60 mK, *f*_Q_ reaches a value of 8.2 ± 0.9 (Supplementary Section D). At a field of 5.8 T, far away from the QCP, *f*_Q_ is strongly suppressed (Supplementary Section H and Supplementary Fig. 11), underpinning the interpretation that Kondo destruction quantum criticality creates high multipartite entanglement. To evaluate the entanglement depth associated with a given *f*_Q_, one has to specify the type of interaction between the neutron and the sample. For scattering from localized spins, with a minimum and maximum expectation value ${h}_{\min }$ and ${h}_{\max }$ of the operator appearing in *S*(**q**, *ω*, *T*), the system must be at least (*m* + 1)-partite entangled if *f*_Q_ satisfies the bound ${f}_{{\rm{Q}}} > m{({h}_{\max }-{h}_{\min })}^{2}$, where *m* is an integer^21,36^. Thus, the normalized QFI^35^

5 $${\rm{nQFI}}=\frac{{f}_{{\rm{Q}}}}{{({h}_{\max }-{h}_{\min })}^{2}}$$

witnesses at least (*m* + 1)-partite entanglement if nQFI > *m*. Previous work has focused on the case of localized spin-1/2 systems^22,35^, where ${({h}_{\max }-{h}_{\min })}^{2}=c{g}^{2}{[(+1/2)-(-1/2)]}^{2}=c{g}^{2}$. *c* counts the spin directions that are probed by *S*(**q**, *ω*, *T*) (refs. ^22,37^) and *g* is the Landé factor.

As described above, at the QCP of Ce_3_Pd_20_Si_6_ we study here, local quantum critical fluctuations derive from the destruction of the Kondo screening of electric quadrupoles (Fig. 1)^24^, made visible to the neutrons through the secondary magnetic dipole moments ${\mu }_{\sec }$ induced by the applied magnetic field (along its direction) on top of the primary electric quadrupole moments. For Ce_3_Pd_20_Si_6_, with a Landé factor *g* = 1 (ref. ^38^), *c* = 1 because the secondary moments are *B* induced, and assuming that the size of the field-induced moment corresponds to a full Bohr magneton *μ*_B_, we obtain nQFI = 8.2 ± 0.9. Any ratio $r={\mu }_{\sec }/{\mu }_{{\rm{B}}} < 1$ will boost nQFI as nQFI/*r*^2^ (Supplementary Section E). As nQFI provides—by its very nature^21^—a lower bound for multipartite entanglement, the present results witness a state with at least 9-partite entanglement. Note that a reduction of *r* from 1 is only one of several reasons why this lower bound of multipartite entanglement is conservative (Supplementary Section I).

We now describe auxiliary-field quantum Monte Carlo simulations (Supplementary Section F) of a Kondo destruction transition and compare them with our experimental results. As a (sign-problem-free) model, we use a spin-1/2 Heisenberg chain on a two-dimensional Dirac semimetal akin to graphene^10^. The exchange interaction among the local moments of the spin chain competes with the Kondo coupling *J*_K_ of the local moments to the conduction electrons, which possess a pseudogap^39^. In the Kondo-screened phase at large *J*_K_, a new particle described by the composite fermion operator ${\widehat{\Psi }}_{{\bf{i}}}^{\dagger }$ (ref. ^40^) emerges. It carries the quantum numbers of the electron and participates in the Luttinger volume such that this state can be identified as the heavy-fermion phase with a large Fermi surface. In the Kondo destruction phase at low *J*_K_, the composite fermion spectral function is purely incoherent (Supplementary Section F and Supplementary Fig. 7). The transition between these two regimes is driven by charge degrees of freedom and a sudden change in the Luttinger volume count at zero temperature, which are clear signs of Kondo destruction physics.

The differences between our model and material are obvious (Supplementary Section F), but what they share is the presence of a Kondo destruction QCP. This, together with the unbiased nature of the simulations, allows us to assess whether the enhanced QFI found in the experiment is a generic feature of Kondo destruction transitions.

We define two different types of QFI: one for the (bosonic) spin fluctuations denoted as *f*_Q_, defined as in equation (1), and one for the composite fermions ${\widehat{\Psi }}_{{\bf{i}}}^{\dagger }$, denoted as ${f}_{{\rm{Q}}}^{\,\Psi }$. The latter is related to the single-particle spectral function *A*(*q*, *ω*) of the composite fermion via

6 $${f}_{{\rm{Q}}}^{\Psi }(T)=2{\int }_{-\infty }^{\infty }{\tanh }^{2}\left(\frac{\hslash \omega }{2{k}_{{\rm{B}}}T}\right)A(q,\omega ){\rm{d}}(\hslash \omega ).$$

Even though this quantity is not ideal for detecting multipartite entanglement—it is bounded for all wavevectors by a sum rule for ∫*A*(*q*, *ω*)d*ω* at *T* = 0 (Supplementary Section F and ref. ^40^)—its temperature dependence provides valuable information. This is because the temperature corrections to the sum rule at the Fermi wavevector *q*_F_ = π/2 depend on the nature of the spectral function.

In the Kondo destruction phase (small *J*_K_), the spins are decoupled from the conduction electrons. In our specific model, *f*_Q_ matches that of an isolated spin-1/2 chain, with critical AFM spin fluctuations at the wavevector *q* = π (ref. ^34^). The composite fermions are purely incoherent, leading to a small and only weakly temperature-dependent ${f}_{{\rm{Q}}}^{\,\Psi }$. In the Kondo-screened phase (large *J*_K_), *f*_Q_ is suppressed and saturates at low temperatures. ${f}_{\rm{Q}}^{\,\Psi }$ near the Fermi wavevector *q*_F_ shows *T*^2^ corrections to the sum rule as expected for a heavy Fermi liquid, and a *T*^3^ law away from it (Supplementary Section F and Supplementary Figs. 8 and 9 show details on both these phases).

**Fig. 4:**
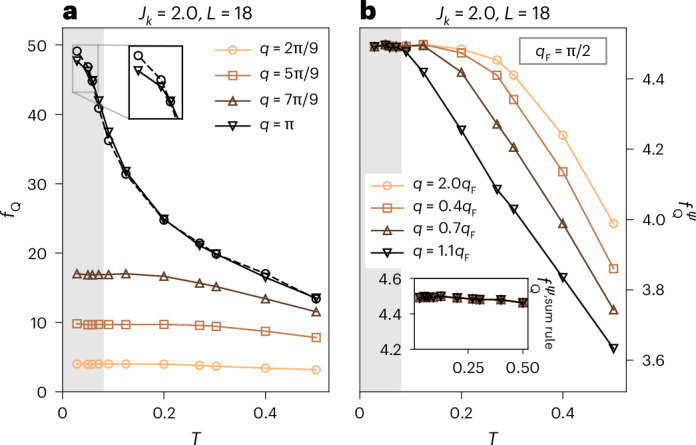
Quantum Monte Carlo simulations of the QFI at the Kondo destruction transition. **a**, QFI density *f*_Q_ for the spin degree of freedom at the wavevector *q* = π of AFM fluctuations and at several wavevectors away from it. At *q* = π, *f*_Q_ grows substantially in the low-temperature limit, but at other vectors, it converges to a finite value. The shaded region indicates where effects due to finite system size set in. The dashed curve is for a larger system (*L* = 22) and tends to saturate only at lower temperatures. As our *f*_Q_ takes into account all three components of spin–spin correlations, we have ${({h}_{\max }-{h}_{\min })}^{2}=3{g}^{2}=12$ in equation (5), and hence, nQFI = *f*_Q_/12. **b**, QFI density ${f}_{{\rm{Q}}}^{\,\Psi }(T)$ for the composite fermion. Wavevectors closer to the Fermi surface *q*_F_ = π/2 show the most pronounced temperature dependence, reflecting the spectral weight corresponding to the heavy-fermion quasiparticle. The shaded area has the same meaning as in **a**. Inset: wavevector-independent and weakly temperature-dependent contribution to ${f}_{{\rm{Q}}}^{\,\Psi }(T)$ from the sum rule (Supplementary Section F). The error bars (smaller than the symbol sizes) result from statistical errors of the Algorithms for Lattice Fermions library^52^ implementation of stochastic maximum entropy calculations^53–55^ used to obtain the QFI from imaginary-time data obtained in quantum Monte Carlo simulations. Source data

We now describe the characteristics of *f*_Q_ and ${f}_{{\rm{Q}}}^{\,\Psi }$ at the critical value of *J*_K_. The value of *f*_Q_ at the wavevector *q* = π increases strongly with decreasing temperature, without a characteristic scale (Fig. 4a), reaching values much enhanced compared with the Kondo-screened phase (though still limited by the finite system size of our simulations; Fig. 4a, shaded area). This reflects the destruction of the composite fermion operator and the concomitant liberated critical spin degrees of freedom. The scale-free increase in *f*_Q_ and the large values reached at the lowest temperatures are common characteristics of our simulations and experiments, and we, thus, identify them as signatures of Kondo destruction quantum criticality. The saturation of *f*_Q_ seen for wavevectors away from *q* = π is due to the fact that the Kondo destruction quantum criticality is not local in our model; this discrepancy with the experiment conforms to our expectation and further underpins the above result. Regarding the composite fermions, ${f}_{{\rm{Q}}}^{\,\Psi }$ at the Fermi wavevector *q*_F_ increases strongly with decreasing temperature (Fig. 4b), and only saturates at the lowest temperature due to the femionic sum rule (Fig. 4b, inset). Outside the saturation region (Fig. 4b, shaded area), we find an approximately linear temperature dependence. This agrees with the theoretically expected linear-in-temperature correction from the smooth incoherent part of the Green’s function at the Kondo destruction transition, which dominates the spectral weight at *q*_F_ (Supplementary Section F), and reflects the loss of quasiparticles evidenced by shot noise experiments^6^.

A few comments are due on the Kondo destruction side of the QCP. Here the spin degrees of freedom can form various phases of matter, both with and without long-range order^2,41^, but this does not change the behaviour at the QCP. For instance, changing the symmetry from SU(2) to SU(4) (antisymmetric self-adjoint representation) in our model calculations will frustrate the AFM interactions, but should not alter the overall behaviour of the QFI at criticality in both spin and single-particle channels. Within the extended dynamical mean-field theory description of Kondo destruction quantum criticality^7^, the QFI peaks at the QCP^42^. The rise of *f*_Q_(*T*) without a scale^42^ is similar to what we find here.

**Fig. 5:**
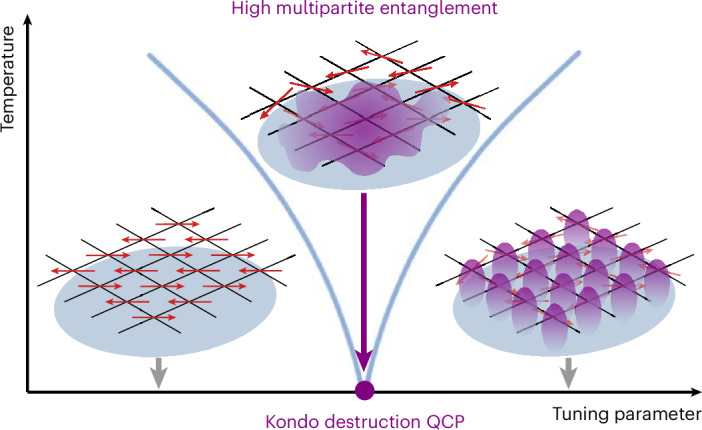
Visualization of enhanced multipartite entanglement in the Kondo destruction scenario. Schematic of the phase diagram of the temperature-tuning parameter, exhibiting a Kondo destruction QCP, which separates a Kondo destruction phase to its left from a Kondo-screened phase to its right, as addressed previously^2–9^. The red arrows represent spins (or pseudo-spins in the case of the orbital Kondo effect) of localized electrons, and blue shadings indicate the conduction electrons. The Kondo destruction phase to the left of the QCP has no static Kondo screening, and the spins typically (but not necessarily) are in order. Violet shadings indicate Kondo screening. In the Kondo-screened phase to the right of the QCP, the individual Kondo clouds (which may have more complex internal character than sketched) form the heavy quasiparticles. The present work provides evidence that in the strange metal realized in the quantum critical fan emanating from the QCP (delimited by the blue lines), a distinct quantum state with high multipartite entanglement forms.

In summary, the dynamical scaling of the INS data found here (Fig. 2b), together with the previously observed momentum space structure of the critical fluctuations (Fig. 1b)^26^ and transport (Fig. 1c,d) and thermodynamic characteristics^24^ provide evidence that Ce_3_Pd_20_Si_6_ exhibits a Kondo destruction QCP near 1.73 T. In the Kondo destruction scenario^2–9^ (Fig. 5), the strange metal state emerges from such a Kondo destruction QCP, which separates a Kondo destruction phase to its left (at small Kondo coupling) from a Kondo-screened phase to its right (at large Kondo coupling). Our quantum Monte Carlo simulations reveal that in the former (where the spins are decoupled from the conduction electrons), the QFI is model dependent. In the latter, for a Fermi liquid with quasiparticles composed of localized spins and conduction electrons, the entanglement depth measured by *f*_Q_ is small. The question we have addressed is whether the destruction of the composite fermion at the QCP is accompanied by an enhancement in the entanglement depth. Our results, both experimental and numerical—for widely different realizations of the Kondo destruction transition (Supplementary Section F)—show that this is indeed the case. The coherent-to-incoherent transition of the composite fermion that underlies this transition, in which the spectral weight is pushed away from the Fermi energy, is picked up by the QFI. The enhanced multipartite entanglement it witnesses points to the microscopic basis of scale invariance^5^ and the loss of quasiparticles^6^ and, therefore, of linear-in-temperature strange metallicity: a collective action of multiple parties resulting from the quantum superposition of their wavefunctions appears to create this behaviour.

To the best of our knowledge, the pronounced scale-free increase in the QFI with decreasing temperature, as observed in our INS investigation of a stoichiometric heavy-fermion compound, points to the largest entanglement depth reported so far in any quantum material, including the proximate quantum spin liquid material KYbSe_2_ above its Néel temperature^43^. This is particularly noticeable because, unlike in KYbSe_2_, we probed the QFI away from any magnetic ordering wavevector, clearly evidencing the beyond-order-parameter nature of the underlying quantum criticality. Disorder effects can be ruled out as away from the QCP, the system behaves as a normal Fermi liquid. Whether high multipartite entanglement is a universal property of strange metals is a question of central importance, and we hope that our work will motivate QFI experiments across the different strange metal platforms^1^. If so, this would open a constructive avenue for scrutinizing the strange metal problem.

Because the strength of quantum critical fluctuations increases steeply with decreasing temperature and energy, such experiments should be performed on spectrometers with the highest possible energy resolution at the lowest possible temperatures and be combined with careful background measurements (Supplementary Section G and Supplementary Fig. 10 show the case of CeCu_5.9_Au_0.1_ (ref. ^4^)). Methods based on inelastic X-ray scattering^44^, angle-resolved photoemission spectroscopy^45^ and electron energy-loss spectroscopy^46^ have recently been proposed as alternatives to probe the QFI. Apart from challenges to quantify their responses and, thus, determine the QFI in absolute units, their energy resolution and the lowest accessible temperatures are still orders of magnitude away from what can be achieved with state-of-the-art INS experiments; as such, these techniques are limited to systems with large intrinsic energy scales.

We anticipate that our work will boost the dialogue with the quantum information science community. On one hand, to fully understand strange metals and related phenomena in other settings such as topological semimetals^47^, further experimentally accessible entanglement witnesses, for example, to detect the entanglement structure^48,49^, should be developed. On the other hand, genuine multipartite entanglement is necessary to reach the maximum sensitivity in certain metrological tasks^36,50^, making strange metals interesting material candidates.

## Online content

Any methods, additional references, Nature Portfolio reporting summaries, source data, extended data, supplementary information, acknowledgements, peer review information; details of author contributions and competing interests; and statements of data and code availability are available at 10.1038/s41567-026-03298-0.

## Supplementary information

Supplementary Information Supplementary Sections A–I and Figs. 1–11.

## Source data

Source Data Fig. 2 *X*, *Y*, err columns for each curve. Data for panels a and b are labelled with separators; the measurement units are also provided.

Source Data Fig. 3 *X*, *Y*, err columns for each curve.

Source Data Fig. 4 *X*, *Y*, err columns for each curve. Data for panels a and b are labelled with separators.

## Data Availability

All data presented in this Article are available via Zenodo at 10.5281/zenodo.19349955 (ref. ^51^). Source data are provided with this paper.

## Code availability

The numerical calculations were carried out using the Algorithms for Lattice Fermions library^52^.
