# Supplementary material for: Quantum Fisher information in a strange metal
Source: Nat Phys. 2026 Jun 15;22(7):1064–70. doi: 10.1038/s41567-026-03298-0 (PMC13423831; doi:10.1038/s41567-026-03298-0)
Supplement: Supplementary file 1 — Supplementary Sections A–I and Figs. 1–11. [file 41567_2026_3298_MOESM1_ESM.pdf]

nature physics

Supplementary information https://doi.org/10.1038/s41567-026-03298-0

Quantum Fisher information in a strange metal

In the format provided by the authors and unedited

Contents

A. Background on Kondo destruction quantum criticality 2

B. Background on multipartite entanglement 4

C. Neutron scattering experiment and data extraction 6

D. Absolute cross-section normalization of INS data 9

E. How neutrons detect quadrupole moments 15

F. Quantum Monte Carlo simulations 19

G. Comparison with $CeCu_{5.9}Au_{0.1}$ 24

H. Measurement of $Ce_3Pd_{20}Si_6$ at 5.8 T 26

I. Why 9-partite entanglement is a conservative lower bound 27

References **29**

A. Background on Kondo destruction quantum criticality

Kondo destruction or local quantum criticality was put forward by analyzing a Kondo lattice model

$$H = \sum_{ij,\sigma} t_{ij} c^{\dagger}_{i\sigma} c_{j\sigma} + \sum_i J_{\mathrm{K}} \boldsymbol{S}_i \cdot \boldsymbol{s}_{c,i} + \sum_i I_{ij} \boldsymbol{S}_i \cdot \boldsymbol{S}_j \quad , \tag{S1}$$

which describes the coupling of spin $1/2$ moments $\boldsymbol{S}_i$ on lattice sites i with conduction electrons, within extended dynamical mean field theory (EDMFT) (*1*). This treatment calculates the local correlation functions of the lattice model in terms of an effective single-site problem, with the electron and spin self-energies taken to be momentum independent. The fluctuations in the spin and charge sectors are kept track of through bosonic and fermionic dissipative baths that are determined self-consistently by the corresponding local correlation functions. Furthermore, by varying the form of the spin-spin coupling in the last term of Eqn. S1, which is mediated by conduction electrons (RKKY type interaction), different types of quantum critical points can be realized. When the RKKY density of states increases from zero at the lower band edge with a jump, the quantum critical point is of the Kondo destruction type. If, by contrast, this increase occurs in a square-root fashion, the asymptotic low-energy form of the dynamical spin susceptibility is as in the standard (Hertz-Millis type (*2, 3*)) description of quantum criticality. For the Kondo destruction case, the dynamical spin susceptibility takes the form

$$\chi(\boldsymbol{q}, \omega, T) = \frac{1}{f(\boldsymbol{q}) + AT^{\alpha} W(\hbar\omega / k_{\mathrm{B}} T)} \ , \tag{S2}$$

where $W(\hbar\omega/k_{\mathrm{B}}T)$ is a scaling function whose form depends only on the anomalous exponent α, that is fractional (*4*). The function $f(\boldsymbol{q}) = I_{\boldsymbol{q}} – I_{\boldsymbol{Q}}$ captures the RKKY interaction, with $I_{\boldsymbol{q}}$ being the Fourier transform of I_{ij} in Eqn. S1. $f(\boldsymbol{q})$ is zero at the (AFM) ordering wavevector $\boldsymbol{Q}$ of a phase that is suppressed at the quantum critical point, varies as $(\boldsymbol{q} - \boldsymbol{Q})^2$ for $\boldsymbol{q}$ close to $\boldsymbol{Q}$,

and changes smoothly for $\boldsymbol{q}$ far away from $\boldsymbol{Q}$ (*1*). At $\boldsymbol{q} = \boldsymbol{Q}$, Eqn. S2 thus reduces to

$$\chi(\boldsymbol{Q}, \omega, T) = \frac{1}{AT^{\alpha} W(\hbar\omega/k_{\mathrm{B}}T)} \, . \tag{S3}$$

The fact that this scaling form describes our data so well at the selected wavevector $(0\,\bar{1}\,0)$, which is far away from the AFQ ordering wavevector $(1\,1\,1)$, means that $f(\boldsymbol{q})$ must be small, at least on the scale of the energy resolution of the experiment. The broad intensity distribution on the reciprocal space map of Fig. 1B supports this statement. The underlying physics is that Kondo destruction quantum criticality involves fluctuations between Kondo-screened and unscreened moments, which is a local process. Far away from the ordering wavevector, this process dominates the inelastic neutron signal. A competing process, the (antiparallel) alignment of neighboring moments via the RKKY interaction, ultimately leads to long-range order below the critical field of 1.73 T. Fluctuations of the order parameter at the border of this phase appear in the vicinity of the ordering wavevector, and do thus not have sizable contributions at the wavevector $(0\,\bar{1}\,0)$ selected for the present study.

This theory of Kondo destruction quantum criticality (*1*) was first applied to INS data on the heavy fermion compound $CeCu_6$, substituted with 0.1 Au atom per formula unit to reach a quantum critical point (*5*). More recently, the treatment of the spin $1/2$ case was extended to an SU(4) spin–orbital Kondo state in a multipolar Bose-Fermi Kondo model, which provides an effective description of a multipolar Kondo lattice (*6*). Using a renormalization group method it was shown that, at zero temperature, a generic trajectory in the model's parameter space contains two quantum critical points, associated with the destruction of Kondo entanglement in the orbital and spin channels, respectively. A related single-impurity model, which considers further multipolar local moments and couplings, finds the same kind of two-stage transition (*7*). Together these works provide a solid theoretical basis for understanding the two consecutive magnetic field-induced Kondo destruction quantum critical points found in $Ce_3Pd_{20}Si_6$ (*8, 9*).

At the quantum critical point (QCP) studied in the present work, the spin degrees of freedom of the Γ_8 quartet are Kondo-screened and remain so across the transition. Thus, they do not contribute to the quantum critical fluctuations. By contrast, the Kondo screening of the electric quadrupole moments breaks up, leading to Kondo destruction-type quantum critical fluctuations of orbital moments. As this happens at a finite magnetic field—this QCP is situated at 1.73 T (*9*)—these fluctuations become visible to neutrons via small magnetic dipole moments that the field induces on top of the orbital moments (*10*). These secondary dipole moments undergo the same quantum fluctuations as the primary quadrupole moments and thus act as their magnetic markers. Note that an investigation of the momentum space structure of the dynamical spin susceptibility upon approaching the ordering wavevector $(1\,1\,1)$ of the AFQ phase—expected from Eqn. S2 and studied in detail in pioneering work on $CeCu_{5.9}Au_{0.1}$ (*5*)—is a topic for future experiments. As opposed to $CeCu_{5.9}Au_{0.1}$, the momentum structure $f(\boldsymbol{q})$ in $Ce_3Pd_{20}Si_6$ reflects the underlying microscopic RKKY interaction projected to the quadrupolar sector; this makes it an interesting problem also for future materials specific theoretical work.

B. Background on multipartite entanglement

Entanglement, a fundamental concept in quantum mechanics, describes a special type of quantum correlation within a composite quantum system. Specifically, it refers to systems exhibiting global quantum states that cannot be expressed as the product of the states of individual subsystems (*11*). Here, we are interested in quantum materials, i.e., full many-body quantum systems consisting of the order of 10^{23} atoms. Subsystems can refer to different parts (parties, partitions) thereof, or the individual constituent particles (spins, electrons, etc.). From the perspective of a particle, entanglement implies that its quantum state cannot be described independently of the states of other particles.

The full density matrix of a quantum system encodes all information about the system,

including its entanglement properties. However, determining the full density matrix is feasible only for small systems, using techniques like quantum state tomography. For moderately larger systems, methods involving random unitaries are applied. In the case of full many-body systems, alternative approaches that rely on partial information become necessary. One such approach is the construction of entanglement witnesses (*12*); the QFI used in the present work is one such witness.

The Fisher information and its quantum version, the QFI, are fundamental notions of (quantum) metrology. They quantify the precision of parameter estimation. Quantum states with particles exhibiting certain forms of quantum entanglement, in particular multipartite entanglement, have been shown to provide higher precision than an ensemble of uncorrelated particles; they are said to exhibit sub-shot-noise precision, which means that the measurement uncertainty scales better than in the classical limit (*13*). Notably, for a system with N particles, the achievable precision $\Delta\Theta$ in the estimation of the parameter Θ scales as $(\Delta\Theta)^2 \propto 1/N$ for nonentangled particles, but can reach $(\Delta\Theta)^2 \propto 1/N^2$ for entangled particles. Consequently, the QFI is bounded by $F_\mathrm{Q} \leq N$ in the first and by $F_\mathrm{Q} \leq N^2$ in the second case (*13*). Finding and preparing quantum states with maximal QFI in a given scenario is hence the key to improving the sensitivity of a quantum measurement device. From the perspective of characterizing quantum materials, a large QFI evidences situations in which the quantum state reacts particularly sensitively to a parameter change.

A key result enabling the present work is that the QFI is measurable even in a full many-body system, under the sole condition that it is in thermodynamic equilibrium (*14*). As explained in the main text, this is due to the rigorous relation between the QFI and a Kubo response function that characterizes the same thermal state and operator for which the QFI is evaluated (Eqn. 1). Because the imaginary part of the dynamical susceptibility $\chi''(\omega, T)$ that probes this response relates, through the fluctuation-dissipation theorem, to the dynamical spin correlation function

$S(\boldsymbol{q}, \omega, T)$, INS experiments can be used to measure the QFI. The entanglement depth, which can be determined from the QFI, quantifies the extent of entanglement: It refers to the minimum number of entangled particles (or parties) in the quantum system. A depth of m means that there is at least one set of m entangled particles (or parties) in the total quantum system.

Multipartite entanglement plays an important role in quantum technologies, including quantum computing, communication, sensing, and metrology. For instance, multipartite entanglement is needed for performing certain quantum algorithms, and a deeper entanglement can lead to more precise measurements. On the other hand, knowledge of the entanglement depth is a first step in describing the structure of quantum states. As such, it is a powerful tool to advance the microscopic understanding of exotic quantum phases, notably the strange metal state.

C. Neutron scattering experiment and data extraction

We performed the neutron scattering experiment at the cold-neutron triple-axis spectrometer ThALES (ILL, Grenoble, France) (*15*). As a reactor-based neutron source, ILL delivers an extremely stable, continuous neutron flux, which is ideal for the type of experiments performed here (long counting at fixed conditions, normalization-sensitive measurements, experiments relying on absolute intensities). Two large single crystals of $Ce_3Pd_{20}Si_6$ with a total mass of 5.9 g (*16*) (corresponding to a volume of 0.605 cm^3) were co-aligned and mounted on a copper sample holder in the $(H\,K\,0)$ scattering plane, with $[0\,0\,1]$ as the vertical axis. Note that the directions x, y, and z along the principal axes of the cubic crystal are defined via the mounting of the crystal on the sample holder; once defined, this notation is kept through all measurements using this same mounting. This is why for the measurements at 1.73 T and the additional measurements at 5.8 T (Sect. H) the scattering wavevector takes different forms, $(0\,\bar{1}\,0)$ and $(1\,0\,0)$, respectively, though these directions are of course equivalent. The sample was placed in a ^{3}He/^{4}He dilution refrigerator inside a 2.5 T vertical magnet. Thus, the magnetic field is

parallel to the $[0\,0\,1]$ crystallographic direction and the relation $\boldsymbol{q} \perp \boldsymbol{B}$ is preserved for any chosen initial and final wavevector k_i and k_f, respectively. We operated the spectrometer with a fixed $k_\mathrm{f} = 1.3\,\text{Å}^{-1}$ and used a cold Beryllium filter to reduce contamination of the neutron beam. For our experimental configuration, ThALES delivers a neutron flux of approximately 6×10^7 neutrons/(s cm^2).

Before any data analysis, several corrections of the raw data have to be made. Firstly, the incoherent scattering contribution (due to the neutron-nuclei interaction) from the instrumental setup was subtracted from all raw data sets. For this purpose, the sample was removed from the cryostat after the experiments, and the empty setup was measured at the base temperature of the cryostat under the same experimental conditions, in particular at the same position relative to the probed wavevector $\boldsymbol{q} = (0\,\bar{1}\,0)$.

Secondly, neutron absorption by the sample has to be taken into account. The absorption depends on the flux of the incident neutrons and, as the neutron flux varies across the energies probed (we operate at fixed k_f and thus varying incident wavevector k_i), we performed an energy-dependent absorption correction. To make the necessary calculation, we used the volume of the sample, the neutron beam dimension according to the openings of the slits and the absorption cross-section of the single elements tabulated in (*17*), revised for a cold-neutron spectrometer. We found the absorption to vary from 10.5% at 0 meV to 8.63% at 1 meV and corrected the data with a smooth fitting function between these values.

Finally, the incoherent elastic contribution from the sample itself has to be identified and subtracted. As this is done together with the analysis of the magnetic scattering, we describe it in Sect. D.

Our study aims at detecting fluctuations from a Kondo destruction QCP. We have therefore probed the system at the quantum critical magnetic field of 1.73 T, away from any magnetic Bragg peak, where order-parameter fluctuations are expected to intervene. As can be seen from

the previously taken time-of-flight data (*18*) shown in Fig. 1B (at 50 mK, intensity integrated from 0.15 meV to 0.35 meV and within ± 0.08 r.l.u. in the orthogonal momentum direction), the intensity is distributed broadly in momentum space. This is a characteristic of Kondo destruction quantum criticality (see Ref. 5 for an early example): fluctuations are due to the dynamical process of the Kondo cloud forming and breaking up, which is local in real space. This is in contrast to quantum phase transitions dominated by order-parameter fluctuations, where fluctuations are due to moments trying to align with their neighbors and intensity appears near the ordering wavevector. At fields away from the quantum critical field, the broad momentum space distribution of low-energy intensity is absent (Fig. S1), confirming that it arizes from the QCP at 1.73 T.

To get a feeling for the momentum space structure of the quantum critical fluctuations, we performed additional experiments at ThALES (*19*), along the two trajectories indicated in Fig. 1B. As shown in Fig. S2, the integrated intensity (from 0.15 meV to 0.35 meV; corresponding to that displayed on the map of Fig. 1B) varies both along the line of equal radius (i.e. for the same absolute value of the scattering wavevector, $|\mathbf{q}|$, panel A) and the one with varying $|\mathbf{q}|$ (panel B). On this latter trajectory, the observed intensity variation is much more pronounced than that of the magnetic form factor of Ce (*20*), meaning that the intensity of the quantum critical fluctuations does have distinct momentum dependence. This is in agreement with the EDMFT result for Kondo destruction quantum criticality. As discussed in Sect. A, the maximum intensity of local quantum fluctuations is expected at the ordering wavevector, even though the quantum criticality is of beyond order parameter fluctuation-type. By collecting data away from it, we derive a lower bound for the QFI (see Sect. I).

D. Absolute cross-section normalization of INS data

Whereas dynamical scaling analyzes such as shown in Fig. 2B could, in principle, be performed on INS data in arbitrary units, information about the multipartite entanglement requires the determination of the experimentally detected magnetic scattering intensities in absolute units. Here we use the method of normalization with sample incoherent elastic scattering (*21*). As discussed at the end of this section, it is the most reliable method for our setup.

Incoherent elastic scattering background subtraction

Incoherent elastic scattering is due to the interaction of the neutrons with the nuclei of the sample. It is included in all data sets obtained as described in Sect. C and must be separated from the inelastic/quasielastic magnetic scattering before the latter can be further analyzed. In addition, the incoherent elastic scattering intensity is used to obtain the data in absolute units. All our measurements were taken at the wavevector $\boldsymbol{q} = (0\,\bar{1}\,0)$, with a magnetic field of 1.73 T applied along the crystallographic $[001]$ direction. There is neither a nuclear nor a magnetic Bragg peak at this wavevector, which is essential for the success of our analysis.

Two exemplary data sets, taken at 85 mK and 10 K and spanning $\hbar\omega = 0$, are shown in Fig. S3. Each curve was fitted by a sum of an incoherent elastic contribution $I_{\mathrm{inc}}(0\,\bar{1}\,0, \omega)$ with a Gaussian line shape

$$f(E) = G \exp\left(-\frac{E^2}{2\sigma^2}\right) \tag{S4}$$

centered around zero energy and with a standard deviation σ fixed to the instrumental resolution (blue curves), two Lorentzian spectral functions for the magnetic response from the sample, and an energy-independet background term that takes care of spurious offsets. The fit (red curves) describes the data very well. For the Gaussian function, the only open fit parameter is the amplitude G.

The energy-integrated incoherent elastic scattering intensity, i.e. the integral of such a Gaus-

sian

$$I_{\rm inc}^{\rm int}(0\,\bar{1}\,0) = \int I_{\rm inc}(0\,\bar{1}\,0,\omega)d\omega\ , \tag{S5}$$

is determined at each temperature, and found to be temperature independent within error bars. This confirms our expectation that the background is temperature-independent at sufficiently low temperatures (10 K and below, where our measurements were performed) and underlines the robustness of the background determination. To reduce unavoidable noise close to zero energy transfer, further analyses of the magnetic scattering were done with the data treated by Gaussian smoothing.

At 85 mK, the integrated coherent intensity is approximately 25% of the total signal; with the used statistics of the measurement, this yields error bars of the QFI of about 10% (see subsection Error determination). To test whether our model describes the data accurately, we analyze the normalized residuals

$$r_i^{\rm norm} = \frac{I_{\rm exp}(\omega_i) - I_{\rm fit}(\omega_i)}{\sigma(\omega_i)}\ . \tag{S6}$$

We find the $r_i^{\rm norm}$ to scatter statistically around zero, confirming that no systematic error is introduced by the fitting procedure. Furthermore, to assess the overall fit quality, we determine the sum

$$\frac{1}{N_\omega}\sum_{i=1}^{N_\omega} r_i^{\rm norm}\ , \tag{S7}$$

where N_ω is the total number of energy data points. For the data at 85 mK, this sum is 2.33×10^{-2} over the probed energy range.

Normalization via incoherent elastic scattering

We now describe the normalization process via incoherent elastic scattering. The mean values over all temperatures (up to 10 K) of the Gaussian amplitude G and the integrated intensity $I_{\rm inc}^{\rm int}(0\,\bar{1}\,0)$, denoted as $\overline{G}$ and $\overline{I_{\rm inc}^{\rm int}}(0\,\bar{1}\,0)$, respectively, are given in Table S1. Frequently, the

intensities in terms of detector counts are divided by the monitor counts; however, here we have taken all data with the same monitor counts (two measurements, each with 2.5 million neutrons on monitor 1) and therefore do not need to do this normalization. Next, we have to estimate the cross-section for incoherent elastic scattering coming from one unit cell of our material, which is

$$\left.\frac{d\sigma}{d\Omega}\right|_{\text{inc}}^{\text{el}} = \frac{1}{4\pi}\sum_j \sigma_j^{\text{inc}} e^{-2W_j} \ , \tag{S8}$$

where j runs over all atoms in the unit cell, which contains four $Ce_3Pd_{20}Si_6$ formula units, σ_j^{inc} is the incoherent neutron scattering cross-section of the j-th atom, and e^{-2W_j} is the Debye-Waller factor of the j-th atom. To evaluate this expression, we assume $e^{-2W_j} = 1$, which is a good approximation at sufficiently low temperatures ($\leq 10\,\text{K}$), and use the tabulated values (*17*) $\sigma_{\text{Ce}}^{\text{inc}} = 0.001$, $\sigma_{\text{Pd}}^{\text{inc}} = 0.093$, $\sigma_{\text{Si}}^{\text{inc}} = 0.004$, all in barn (b).

The resolution volume (*21*)

$$R_0 = \frac{I_{\text{inc}}^{\text{int}}(0\,\bar{1}\,0)}{\left.\frac{d\sigma}{d\Omega}\right|_{\text{inc}}^{\text{el}}} \tag{S9}$$

connects Eqns. S5 and S8 via the general definition of the neutron scattering intensity

$$I(\boldsymbol{q}, E) = \int \frac{d^2\sigma}{d\Omega_0 dE_0} R(\boldsymbol{q}_0, E_0, \boldsymbol{q}, E) d\boldsymbol{q}_0 dE_0 \ , \tag{S10}$$

where $R(\boldsymbol{q}_0, E_0, \boldsymbol{q}, E)$ is the instrument resolution function, which takes the value R_0 when integrated over energy at the wavevector $(0\,\bar{1}\,0)$ we probed.

Finally, the dynamical spin correlation function per site (or moment) expressed in absolute units can be shown to be given as (*21*)

$$S(\boldsymbol{q}, \omega) = \frac{13.77(\text{b}^{-1}) I(\boldsymbol{q}, E)}{|f(\boldsymbol{q})|^2 e^{-2W} R_0} \times \frac{8}{12} \,, \tag{S11}$$

where $f(\boldsymbol{q})$ is the magnetic form factor of Ce at the scattering wavevector $\boldsymbol{q}$ = $(0\,\bar{1}\,0)$ probed in our experiments and the ratio $8/12$ reflects the fact that out of the 12 Ce atoms per unit cell only the 8 ones at the $8c$ site are magnetically active (*22*). We used $|f(\boldsymbol{q})|^2 = 0.984$, as calculated

in (*20*), which is in good agreement with experiments on both γ-Ce (*23*) and α-Ce (*24*), and calculations for free Ce^{3+} ions (*23*). $S(\boldsymbol{q},\omega)$ has the unit of 1/meV/Ce_{8c} atom and is the quantity used in the plots in Fig. 2.

Error determination

All steps in this analysis are subject to uncertainties, which lead to the error bars of f_{Q} shown in Fig. 3. The statistical error on the data is estimated as $\Delta I = \sqrt{I}$, where the intensity I is given by the number of counts on the neutron detector, for a given measurement (i.e., at a given energy E, temperature T, momentum $\boldsymbol{q}$). This holds for the total measured intensity I_{total} and the empty cryostat intensity I_{empty} measured with the sample removed. The error on the sample intensity $I_{\mathrm{sample}} = I_{\mathrm{total}} - I_{\mathrm{empty}}$ is then given by

$$\Delta I_{\mathrm{sample}} = \sqrt{(\Delta I_{\mathrm{total}})^2 + (\Delta I_{\mathrm{empty}})^2} = \sqrt{I_{\mathrm{total}} + I_{\mathrm{empty}}}\,. \tag{S12}$$

I_{sample} is then absorption corrected as $I^{\mathrm{corr}}_{\mathrm{sample}} = I_{\mathrm{sample}} \cdot A$, where an absorption correction factor $A = 1.1$ represents an absorption of 10%, and the error in this factor ΔA will contribute as

$$\frac{\Delta I^{\mathrm{corr}}_{\mathrm{sample}}}{I^{\mathrm{corr}}_{\mathrm{sample}}} = \sqrt{\left(\frac{\Delta I_{\mathrm{sample}}}{I_{\mathrm{sample}}}\right)^2 + \left(\frac{\Delta A}{A}\right)^2}\,. \tag{S13}$$

To obtain the intensity I of the quantum critical signal of interest to us here, the incoherent elastic intensity I_{inc}, determined as explained above, must be subtracted. Its statistical error is generally also assumed to be given by $\sqrt{I_{\mathrm{inc}}}$. Then, the total error of the intensity of the quantum critical signal is

$$\Delta I = \sqrt{(\Delta I^{\mathrm{corr}}_{\mathrm{sample}})^2 + (\Delta I_{\mathrm{inc}})^2} = \sqrt{I^{\mathrm{corr}}_{\mathrm{sample}} + I_{\mathrm{inc}}}\,. \tag{S14}$$

The neutron intensities are then brought into absolute units using Eqn. S11, and the errors are propagated according to

$$\frac{\Delta S(\boldsymbol{q},\omega)}{S(\boldsymbol{q},\omega)} = \sqrt{\left(\frac{\Delta I}{I}\right)^2 + \left(\frac{\Delta |f(\boldsymbol{q})|^2}{|f(\boldsymbol{q})|^2}\right)^2 + \left(\frac{\Delta e^{-2W}}{e^{-2W}}\right)^2 + \left(\frac{\Delta R_0}{R_0}\right)^2}\,, \tag{S15}$$

with the relative error of the resolution volume given by

$$\frac{\Delta R_0}{R_0} = \sqrt{\left(\frac{\Delta(\overline{I^{\text{int}}_{\text{inc}}}(0\,\bar{1}\,0))}{\overline{I^{\text{int}}_{\text{inc}}}(0\,\bar{1}\,0)}\right)^2 + \left(\frac{\Delta(\sum_j \sigma_j^{\text{inc}})}{\sum_j \sigma_j^{\text{inc}}}\right)^2 + \left(\frac{\Delta(e^{-2W})}{e^{-2W}}\right)^2}\,. \tag{S16}$$

The error of the first term was determined as follows: (i) the statistical error of a given measurement (at a given energy and temperature), with N counts on the detector, is assumed to be $\sqrt{N}$; (ii) at each temperature, the energy dependence $N(E)$ with these error bars is fitted with a Gaussian, two Lorentzians, and a constant offset. The χ^2 minimization produces error bars for all fit parameters, in particular ΔG for the amplitude G of the Gaussian (Eqn. S4), its only free parameter; (iii) the relative error in the integrated intensity of the Gaussian is also $\Delta G/G$, as the integral of a Gaussian, $\sqrt{2\pi}G\sigma$, is proportional to G and the standard deviation σ is fixed to the instrumental resolution. For both the second and third terms, we assumed an error of 2%. The obtained values and errors are summarized in Table S1.

Finally, to obtain f_Q (at the probed wavevector $\boldsymbol{q}$), the integration over energy is performed and the error is estimated via Simpson's rule

$$\Delta f_\text{Q} = \sqrt{\sum_{i=1}^{N} (\Delta S_i \cdot \Delta E_i)^2}\,, \tag{S17}$$

where S_i is the value of $S(\boldsymbol{q}, \omega)$ at a given energy $E_i = \hbar\omega_i$ and ΔE_i the energy separation between the measured data points.

Alternative normalization methods

Various other methods can be used to determine the resolution volume, but, as we argue here, the approach described above is deemed most accurate in the present situation. Normalization via nuclear Bragg scattering requires a dedicated neutron diffraction experiment, normalization via phonons more energy and momentum coverage and measurements at higher temperatures than achievable here. Neither would yield corrections that are directly applicable to the measured data. However, to test the robustness of our normalization procedure, we measured (i) a

vanadium standard sample and (ii) our $Ce_3Pd_{20}Si_6$ sample outside the cryostat, both at 300 K. In (i), a broad tail of diffuse scattering at low energy transfers complicates the analysis: the data cannot be described by a single Gaussian but had to be fitted with multiple functions. Furthermore, less data acquisition time was dedicated to this measurement. Both together increased the total error bar ΔG considerably. In (ii), we performed an energy scan on the wavevector $(0\,\bar{1}\,0)$. To compare the results with our low-temperature data measured in the cryostat, corrections for the magnet and cryostat transmittance are needed in addition to the absorption correction of Eqn. S13, adding an error that we estimate as 5%. Furthermore, as both (i) and (ii) are done at 300 K, a temperature correction factor α $(10\% \pm 1\%)$ must be applied. The results are summarized in Table S2. Our method is most reliable and precise because it uses the sample itself in the exact same setting, i.e. at the same temperature, on the same sample holder, in the same spectrometer setting, and in the cryostat. Different conditions add errors. Nevertheless, within error bars, all three methods give the same result for both the integrated incoherent elastic scattering intensities and for the resulting QFI density f_{Q}.

Additional measurements with higher resolution

During a second beamtime (*19*), settings were sought that lead to a further enhancement of the measurement resolution: (i) a sharper resolution function was achieved by choosing the final wavevector $k_{\mathrm{f}} = 1.15\,\text{Å}^{-1}$, and (ii) the cold Beryllium filter was removed to increase the neutron flux and thus improve the statistics. The latter, however, limits the useful energy transfer range to below 1 meV, as spurious higher-energy contributions are not eliminated without the filter. Unfortunately, the cryostat background measurement could only be done without the dilution refrigerator unit, and thus, an additional Gaussian was needed to subtract the remaining instrumental background.

One set of data was taken at the quantum critical field of 1.73 T, at the lowest temperature of 80 mK reached during this beamtime, and analyzed as in Sect. C and D, including the

modelling with Gaussians and Lorentzians to separate the elastic incoherent scattering signal from the coherent quantum critical signal. As seen in Fig. S4A, these two contributions can be neatly separated. The normalized residuals (Eqn. S6) scatter statistically around zero, without a systematic structure, demonstrating that our model captures the data accurately (Fig. S4B). The sum (Eqn. S7) is below 2.05×10^{-2}, corresponding to $\chi^2_{\rm red} = 2.16$. The QFI density for this data set is $f_{\rm Q} = 7.44 \pm 0.58$. The sharper resolution, together with the better statistics, decreased the error bar by about 30% (compared to the result at 60 mK of the main result, i.e. $f_{\rm Q} = 8.2 \pm 0.9$). However, likely a combination of the lower cutoff energy, the extra Gaussian background subtraction, and the higher temperature, lead to a slight decrease (by about 8%) of the absolute magnitude of $f_{\rm Q}$. Overall, however, the two experiments, done during different beamtimes, with different configurations, and with independent analyses, clearly demonstrate reproducibility within the error bars.

For the data taken during this beamtime at a field of 5.8 T, see Sect. H and Fig. S11.

E. How neutrons detect quadrupole moments

Interaction operator in magnetic neutron scattering

How strongly a neutron interacts with (is scattered from) a solid depends on the scattering processes at play. Each process can, in principle, be described by a scattering operator $\hat{\mathcal{O}}$ and the neutron scattering cross-section contains a transition matrix element of this operator $\langle f|\hat{\mathcal{O}}|i\rangle$ between the initial and final states of the neutron. In simple cases, e.g., a neutron scattering from nuclei at fixed positions, $\hat{\mathcal{O}}$ can be easily written down. Of interest to us here is magnetic scattering, which is caused by the interaction of a neutron with the magnetic field $\boldsymbol{H}$ (or induction $\boldsymbol{B} = \mu_0 \boldsymbol{H}$, where μ_0 is the vacuum permeability) associated with the sample

$$\hat{\mathcal{O}} = \hat{\boldsymbol{\mu}}_{\rm n} \cdot \boldsymbol{B} = -1.91304276\, \mu_{\rm N}\, \mu_0\, \boldsymbol{\sigma} \cdot \boldsymbol{H} \,, \tag{S18}$$

where $\hat{\boldsymbol{\mu}}_{\mathrm{n}}$ is the magnetic moment operator of the neutron, $\mu_{\mathrm{N}} = 5.05079 \cdot 10^{-27}$ J/T the nuclear magneton, and $\boldsymbol{\sigma}$ are the Pauli matrices (*25*).

There are multiple ways in which a field $\boldsymbol{H}$ can be produced in a solid. The case that has previously been considered in connection with the QFI is that (unpolarized) neutrons scatter from identical magnetic ions with localized electrons, with spin-only scattering from spin $1/2$ moments (*14, 26–30*). Magnetic atoms can, however, also possess orbital angular momenta. Generally, the dipole approximation is adopted where their contribution vanishes (*31*).

In $Ce_3Pd_{20}Si_6$, in zero magnetic field, orbital order sets in below 0.4 K (Fig. 1A), but this transition is invisible for neutrons (*32*) (Fig. S5). This confirms that the dipole approximation is justified. In a finite magnetic field, however, magnetic scattering sets in and, thus, some field-induced magnetic dipole must exist. Indeed, neutron diffraction experiments have observed a magnetic-field induced broad elastic magnetic signal surrounding the (111) structural Bragg peak, thus identifying the hidden order quadrupolar phase (*32*).

Simple Landau theory for quadrupole moments in a magnetic field

Theoretically, this field induction of secondary dipole moments on top of primary quadrupole moments can be rationalized on the level of a simple Landau theory (*33, 34*). We consider the following simplified situation: The primary order parameter is a pure electric quadrupole tensor Q_{ij}. In zero magnetic field, the ordered phase is characterized by a mean value $\langle Q_{ij} \rangle \neq 0$, but $\langle M_i \rangle = 0$, where M_i denotes the magnetic dipole moment. Importantly, M_i is not an order parameter of the quadrupolar phase transition but a noncritical response variable.

The symmetry-allowed Landau free energy in the presence of a magnetic field H_i reads

$$F[Q, M] = \frac{r_Q(H)}{2} Q_{ij} Q_{ij} + \frac{\mu_0}{2\chi_0} M_i M_i - \mu_0 M_i H_i + \mu_0 \lambda Q_{ij} M_i H_j + \dots \, , \tag{S19}$$

where the stiffness $r_Q(H)$ of the primary (quadrupolar) order parameter Q_{ij} is tuned by the magnetic field, M_i describes noncritical dipolar degrees of freedom with a finite bare suscep-

tibility χ_0, and the bilinear term $\mu_0 \lambda Q_{ij} M_i H_j$ is the lowest-order symmetry-allowed coupling that mixes quadrupolar and dipolar sectors in finite field. Here and in the following, summation over repeated indices ($i = x, y, z$) is implied.

Since M_i is noncritical, it can be eliminated by minimizing F with respect to M_i, i.e.,

$$\frac{\partial F}{\partial M_i} = 0 \quad \Rightarrow \quad M_i = \chi_0 \left(H_i - \lambda Q_{ij} H_j \right) \ . \tag{S20}$$

Substituting this expression for M_i into Eqn. S19 yields an effective free energy depending only on the quadrupolar order parameter,

$$F_{\text{eff}}[Q] = \frac{r_Q^{\text{eff}}(H)}{2} Q_{ij} Q_{ij} - \frac{\mu_0 \chi_0}{2} H_i H_i + \mu_0 \chi_0 \lambda H_i Q_{ij} H_j + \ldots \tag{S21}$$

with

$$r_Q^{\text{eff}}(H) = r_Q(H) - \mu_0 \chi_0 \lambda^2 H_j H_j \ . \tag{S22}$$

This formulation highlights that in our problem only Q_{ij} is fundamental, that H_i couples quadratically to Q_{ij}, and that the dipoles M_i appeared only as intermediate bookkeeping. The first term of Eqn. S21 is the primary source of critical fluctuations; it controls the divergence of the quadrupolar susceptibility and dominates the low-energy dynamics near the quadrupolar QCP. The second term, which is independent of Q_{ij}, represents the analytic magnetic background energy of a paramagnet. It is non-singular across the quadrupolar QCP and unrelated to symmetry breaking and critical fluctuations. The third term produces a static, field-induced quadrupole that contributes to elastic, but not to inelastic scattering; as such, it is also irrelevant for the INS dynamics investigated here.

Although M_i is noncritical, Eqn. S20 shows that quadrupolar fluctuations induce a secondary dipolar response in finite field,

$$\delta M_i = -\chi_0 \lambda \, Q_{ij} H_j \ . \tag{S23}$$

Note that we here use the order parameter language, which treats Q_{ij} itself as the fluctuating field. Near the quadrupolar QCP, $\langle Q_{ij}\rangle$ vanishes (whereas $\langle M_i\rangle$ remains finite) and thus $Q_{ij} = \langle Q_{ij}\rangle + \delta Q_{ij} = \delta Q_{ij}$. According to Eqn. S23, the magnetic field generates dipolar fluctuations from fluctuations of the quadrupolar order parameter, thereby making a purely quadrupolar mode visible to probes that couple to magnetic dipole moments (such as neutrons). Importantly, the dipole does not constitute an independent fluctuating degree of freedom. Instead, its magnitude and direction are fully determined by the quadrupolar fluctuations and the externally applied field. Consequently, the magnetic response probed by neutrons inherits all of its quantum fluctuations from the quadrupolar sector, without any additional orientational degrees of freedom or symmetry-related averaging.

The inelastic dynamical structure factor

$$S_{ij}^{\mathrm{inel}}(\mathbf{Q},\omega) = \int_{-\infty}^{\infty} dt\, e^{i\omega t}\, \langle \delta M_i(\mathbf{Q},t)\, \delta M_j(-\mathbf{Q},0)\rangle \quad \text{(S24)}$$

$$\propto H_k H_l \int_{-\infty}^{\infty} dt\, e^{i\omega t}\, \langle Q_{ik}(\mathbf{Q},t)\, Q_{jl}(-\mathbf{Q},0)\rangle \quad \text{(S25)}$$

and, by extension, the QFI therefore directly measure the dynamical quadrupolar correlation function multiplied by H^2.

More advanced, material specific Landau descriptions of magnetic field coupling to quadrupolar order and inducing secondary dipolar responses have, for instance, been developed for the non-Kramers Pr^{+3}: [Xe]$4f^2$ moments in Pr(Ti,V,Ir)$_2$(Al,Zn)$_{20}$ (*35, 36*) and the Γ_8 quartet of Ce^{+3}: [Xe]$4f^1$ in CeB_6 (*37, 38*) and $Ce_3Pd_{20}Si_6$ (*8*).

Estimation of the size of the field-induced secondary dipole moments

Experimentally, the magnetic field induction of dipole moments on top of pure quadrupole moments was used in various compounds to detect hidden order phases (*10, 39–44*), with CeB_6 being the best studied example (*10, 39–41*).

To determine the size of the secondary field-induced dipole moments requires dedicated ex-

periments, such as the combined INS (*40*) and nuclear magnetic resonance (NMR) (*39, 41*) investigations performed in the cubic compound CeB_6 (*10*). As the situation in CeB_6 and $Ce_3Pd_{20}Si_6$ is very similar—also in CeB_6 the Ce^{+3}: [Xe]$4f^1$ electronic configuration assumes a Γ_8 ground state and the system undergoes quadrupolar ordering (*44*)—these results can guide our interpretation. For the configurations investigated in CeB_6 (magnetic field applied along $(1\,1\,1)$ and $(\bar{1}\,1\,0)$), at a field of 1.73 T, the field-induced moment is less than $0.05\mu_\mathrm{B}$ (Fig. S6). For the $(0\,0\,1)$ direction of relevance to us, the moment is 20% smaller than for $(1\,1\,1)$ (*41*), thus about $0.033\,\mu_\mathrm{B}$. Furthermore, the size of the field-induced moment is expected to relate to the rate of increase of the ordering temperature with the field. In CeB_6, it is 0.84 K/T (*44*), about 4 times greater than in $Ce_3Pd_{20}Si_6$ (*9*). This comparison suggests that the moment induced at 1.73 T in $Ce_3Pd_{20}Si_6$ is $< 0.01\ \mu_\mathrm{B}$. In the largest field of 8 T, where the moment size of CeB_6 roughly saturates, the magnitude is about $0.1\,\mu_\mathrm{B}$ (*10, 40, 41*). This moment has neither strong temperature nor strong field direction dependence, suggesting that Kondo screening plays a minor role. We consider this moment to be an upper limit for the field-induced secondary dipole moment in $Ce_3Pd_{20}Si_6$. Using the corresponding moment ratio $r = \mu_\mathrm{sec}/\mu_\mathrm{B} = 0.1$ yields a normalized QFI of $\mathrm{nQFI} = 8.2/(1 \times 1^2)/(0.1^2) = 820$.

F. Quantum Monte Carlo simulations

Unbiased model calculations play an important role in our understanding of exotic quantum phase transitions. The model we discuss in the following is unique since it can be solved numerically exactly and exhibits a Kondo destruction transition. Thus, both our experiments on $Ce_3Pd_{20}Si_6$ and our simulations on the model exhibit the physics of key interest to us here: a continuous quantum phase transition between (i) a phase where a local degree of freedom (orbital moment in $Ce_3Pd_{20}Si_6$ vs spin 1/2 in the model) is decoupled from the conduction elec-

trons (Schrödinger-like electrons in 3D vs Dirac electrons in 2D) and orders (antiferroquadrupolar order in $Ce_3Pd_{20}Si_6$ vs AFM correlations in the model) and (ii) a Kondo screened phase (Kondo-screened orbital moments in $Ce_3Pd_{20}Si_6$ vs Kondo-screened spin 1/2 in the model). The differences between the material and the model help us to understand what is generic to the Kondo destruction transition. As the material and the model have different dimensionalities, scaling laws cannot be expected to be comparable. However, at the Kondo destruction quantum critical point, we find a scale-free increase of the QFI with decreasing temperature in both the material and the model.

We have used the ALF implementation (*45*) of the auxiliary field quantum Monte Carlo algorithm (*46–49*) to simulate a spin-1/2 chain on a semimetallic surface. The Hamiltonian reads

$$\hat{H} = -t \sum_{\langle \boldsymbol{i},\boldsymbol{j} \rangle} \hat{\boldsymbol{c}}^{\dagger}_{\boldsymbol{i}} \, e^{\frac{2\pi i}{\Phi_0} \int \boldsymbol{A}(\boldsymbol{l}) \cdot d\boldsymbol{l}} \, \hat{\boldsymbol{c}}_{\boldsymbol{j}} + \frac{J_{\mathrm{K}}}{2} \sum_{n=1}^{L} \hat{\boldsymbol{c}}^{\dagger}_{n\boldsymbol{a}_x} \boldsymbol{\sigma} \hat{\boldsymbol{c}}_{n\boldsymbol{a}_x} \cdot \hat{\boldsymbol{S}}_n + J_h \sum_{n=1}^{L} \hat{\boldsymbol{S}}_n \cdot \hat{\boldsymbol{S}}_{n+1} \,. \tag{S26}$$

In the above, $\boldsymbol{i}$ runs over the sites of a square lattice with unit vectors $\boldsymbol{a}_x$, $\boldsymbol{a}_y$ and linear length set by La with a the lattice constant. $\hat{\boldsymbol{c}}^{\dagger}_{\boldsymbol{i}} = \left(\hat{c}^{\dagger}_{\boldsymbol{i},\uparrow}, \hat{c}^{\dagger}_{\boldsymbol{i},\downarrow} \right)$ is a two-component spinor of fermion creation operators. The vector potential, $\boldsymbol{A}(\boldsymbol{x}) = -\frac{\pi \Phi_0}{a^2}(y,0,0)$ with the flux quantum Φ_0, accounts for a π-flux that penetrates each plaquette. At this magnetic field, time-reversal symmetry is not broken and the band structure of the conduction electrons is that of Dirac fermions. $\boldsymbol{\sigma}$ is a vector of Pauli spin matrices and $\hat{\boldsymbol{S}}_n$ the spin-1/2 degrees of freedom of the spin chain. At half band filling, this model is free of the negative sign problem such that large-scale computations can be carried out. To access dynamical properties, we have used the stochastic maximum entropy method (*50–52*) implemented in the ALF library (*45*). Our implementation allows us to specify a default model. For the temperature scans used to determine the QFI, we used the higher temperature image as the default model for the lower temperature data. As a non-trivial check, we consider the QFI in the Kondo destruction phase. Previous quantum Monte Carlo

results (*45*) show that in this phase, the spin-1/2 degrees of freedom share the same space- and time-displaced correlation functions as those of the spin-1/2 chain. In Fig. S8 we show the QFI computed as a function of temperature at $J_{\mathrm{K}} = 1$ deep in the Kondo destruction phase. The data support the theoretically expected (*28*) $\log^{3/2}(1/T)$ divergence of the QFI at the wavevector of critical AFM fluctuations. Note that this divergence is specific to the 1D nature of the spin chain: The Mermin-Wagner theorem forbids long-range order in 1D, which produces this divergence. As such, it is not expected for the spins (or orbital moments) in the 3D cubic compound $Ce_3Pd_{20}Si_6$, which do undergo long-range (AFQ) ordering in the Kondo destruction phase.

In Fig. S7 we plot the spectral function of the composite fermion operator

$$\hat{\boldsymbol{\Psi}}_{\boldsymbol{i}}^{\dagger} = \hat{\boldsymbol{c}}_{\boldsymbol{i}}^{\dagger}\, \boldsymbol{\sigma} \cdot \hat{\boldsymbol{S}}_{\boldsymbol{i}}\,, \tag{S27}$$

that has the quantum numbers of the electron (*53*). Here we use the zero-temperature projector version of the auxiliary field quantum Monte Carlo on an 18×18 lattice. Deep in the Kondo-screened phase, at $J_{\mathrm{K}}/t \geq 2.5$, one observes a well-defined quasiparticle pole that crosses the Fermi energy at the one-dimensional momentum $k_x a = \pi/2$. In the Kondo destruction phase, $J_{\mathrm{K}} \leq 1.9$, the quasiparticle loses low-energy weight and becomes incoherent. While the composite fermion spectral function exhibits a coherent to incoherent transition, the conduction electrons remain coherent. The Kondo destruction transition can be understood in terms of an orbital selective Mott transition in which one type of quasiparticle, the composite fermion, localizes and drops out from the Luttinger count. We also supplement the investigations of the QFI at criticality in the main text by displaying the evolution of the QFI across the Kondo destruction transition. In Fig. S8 we display the QFI density for spins, f_{Q}, across the Kondo destruction transition. The QFI in the ordered phase increases with decreasing temperature for the wavevector of critical AFM fluctuations $q = \pi$, whereas it plateaus out in the Kondo-screened phase for all wavevectors. In Fig. S9, we show the composite fermion QFI density f_{Q}^{Ψ}.

We see that wavevectors near the Fermi surface at $q_{\rm F} = \pi$ acquire a pronounced temperature dependence in the Kondo-screened phase, whereas all wavevectors have a weaker temperature dependence in the Kondo destruction phase.

We now consider sum rules and bounds for the bosonic and fermionic QFI. Let us start with a zero-temperature sum rule for the bosonic case, where, for $\hbar = 1$ and $\beta = 1/(k_{\rm B}T)$,

$$f_{\rm Q}(T) = \frac{4}{\pi}\int_0^{\infty} \tanh\left(\frac{\beta\omega}{2}\right)\chi''(q,\omega,T){\rm d}\omega\,. \tag{S28}$$

In the zero-temperature limit and summing over all wavevectors we obtain

$$\frac{1}{N}\sum_q f_{\rm Q}(T=0) = \frac{4}{N}\sum_q \int_0^{\infty} S(q,\omega,T=0){\rm d}\omega = \frac{4}{N}\sum_q \langle \hat{\boldsymbol{S}}(q)\cdot\hat{\boldsymbol{S}}(-q)\rangle\,. \tag{S29}$$

Since $\hat{\boldsymbol{S}}(q) = \frac{1}{\sqrt{N}}\sum_{\boldsymbol{r}} e^{i\boldsymbol{q}\cdot\boldsymbol{r}}\hat{\boldsymbol{S}}_{\boldsymbol{r}}$, the sum rule reads

$$\frac{1}{N}\sum_q f_{\rm Q}(T=0) = 4g^2 s(s+1)\,. \tag{S30}$$

Here $\hat{\boldsymbol{S}}_{\boldsymbol{r}}^2 = g^2 s(s+1)$. At a local quantum critical point, without wavevector dependence, the normalized QFI is bounded in the low-temperature limit. This is consistent with a value of the critical exponent $\alpha < 1$.

While the QFI in the particle-hole channel can take arbitrarily large values in the zero-temperature limit and at a given wavevector, the QFI for fermion operators is necessarily bounded for each momentum. Setting $\hbar = 1$ in Eqn. 6 we obtain

$$f_{\rm Q}^{\Psi}(T) = \frac{2}{\beta}\int_{-\infty}^{\infty} \beta\left(\tanh^2\left(\frac{\beta\omega}{2}\right) - 1\right)A(q,\omega)d\omega + 2\int_{-\infty}^{\infty} A(q,\omega)d\omega\,. \tag{S31}$$

Since for different positions $\boldsymbol{i} \neq \boldsymbol{i}'$ the composite fermion operator anticommutes, the sum rule

$$\int_{-\infty}^{\infty} A(q,\omega)d\omega = \frac{1}{N}\sum_{\boldsymbol{i},s}\left\langle\left\{\hat{\Psi}^{\dagger}_{\boldsymbol{i},s},\hat{\Psi}_{\boldsymbol{i},s}\right\}\right\rangle = -\frac{2}{N}\sum_{\boldsymbol{i}}\left\langle\hat{\boldsymbol{S}}_i\cdot\hat{\boldsymbol{c}}^{\dagger}_{\boldsymbol{i}}\boldsymbol{\sigma}\hat{\boldsymbol{c}}_{\boldsymbol{i}}\right\rangle + 2s(s+1) \tag{S32}$$

with $s = 1/2$ holds. Importantly, it is wavevector independent. By consequence, $f_{\rm Q}^{\Psi}(T=0) = -\frac{4}{N}\sum_{\boldsymbol{i}}\left\langle\hat{\boldsymbol{S}}_i\cdot\hat{\boldsymbol{c}}^{\dagger}_{\boldsymbol{i}}\boldsymbol{\sigma}\hat{\boldsymbol{c}}_{\boldsymbol{i}}\right\rangle + 4s(s+1)$. We now discuss the approach to this zero-temperature value.

Consider the function

$$g_\beta(\omega) = \beta\left(1 - \tanh^2\left(\frac{\beta\omega}{2}\right)\right) . \tag{S33}$$

It is positive and in the low-temperature limit scales to $g_\beta(\omega) = C\delta(\omega)$ with $C = \int_{-\infty}^{\infty}\left(1 - \tanh^2\left(\frac{x}{2}\right)\right) dx$ and $\delta(\omega)$ the Dirac δ-function. With the above,

$$f_{\mathrm{Q}}^{\Psi}(T) - \frac{2}{N}\sum_{\boldsymbol{i},s}\left\langle\left\{\hat{\Psi}^{\dagger}_{\boldsymbol{i},s}, \hat{\Psi}_{\boldsymbol{i},s}\right\}\right\rangle = -\frac{2}{\beta}\int_{-\infty}^{\infty} g_\beta(\omega)A(q,\omega) . \tag{S34}$$

Since both g_β and the spectral function are positive, the bound

$$f_{\mathrm{Q}}^{\Psi}(T) \le f_{Q}^{\Psi,\,\text{sum rule}} = \frac{2}{N}\sum_{\boldsymbol{i},s}\left\langle\left\{\hat{\Psi}^{\dagger}_{\boldsymbol{i},s}, \hat{\Psi}_{\boldsymbol{i},s}\right\}\right\rangle \tag{S35}$$

holds. This has been put forward in Ref. 54 and, as a consequence, the fermion QFI will generically provide a poor bound for multipartite entanglement. However, it provides interesting information on the low-energy behavior of the spectral function. In particular, in the zero-temperature limit, and assuming that at the considered wavevector the spectral function is a smooth function of frequency, we obtain

$$f_{\mathrm{Q}}^{\Psi}(T) = \frac{2}{N}\sum_{\boldsymbol{i},s}\left\langle\left\{\hat{\Psi}^{\dagger}_{\boldsymbol{i},s}, \hat{\Psi}_{\boldsymbol{i},s}\right\}\right\rangle - \frac{2C}{\beta}A(q,\omega=0) + \mathcal{O}\left(\frac{1}{\beta^2}\right) . \tag{S36}$$

This shows that the bound is exhausted in the zero-temperature limit. It also allows for an interpretation of the QFI in terms of UV and IR physics. The sum rule encodes the model-dependent UV physics. Subtracting it from the signal reveals the IR physics; in particular, the value of the spectral function at $T = 0$ and $\omega = 0$ sets the value of the linear-in-temperature correction.

The above assumption of a smooth $A(q,\omega = 0)$ implies the absence of quasiparticles. In a Fermi liquid, $A(q_{\mathrm{F}},\omega) = -\frac{1}{\pi}\mathrm{Im}\frac{1}{\omega+i\Gamma}$ with $\Gamma \propto T^2$. Using this form one can show that the right-hand side of Eqn. S34 scales as T^2.

The numerical results at criticality and at the Fermi wavevector $q_\mathrm{F} = \pi/2$, plotted in Fig. 4B, support a linear-in-T deviation from saturation. As one moves away from the critical point from $J_\mathrm{K} = 2.3$ to $J_\mathrm{K} = 3.0$ one observes a rounding off consistent with a T^2 behavior (Fig. S9). Away from the Fermi energy, and with $A(q, \omega) = -\frac{1}{\pi}\mathrm{Im}\frac{1}{\omega-\omega_q+i\Gamma}$ for $\omega_q \neq 0$, the right-hand side of Eqn. S34 scales as T^3.

In summary, despite distinct differences between the QMC results for our model and phenomena observed in the investigated material $Ce_3Pd_{20}Si_6$, at the critical value of J_K, we have identified the following as signatures of Kondo destruction quantum criticality: (i) a scale-free increase (except for finite-size effects) of the QFI density for the spin degree of freedom $f_\mathrm{Q}(T)$ at $q = \pi$ and large values reached at the lowest temperatures, and (ii) a pronounced increase of the QFI density for the composite fermion $f_\mathrm{Q}^\Psi(T)$ with decreasing temperature close to the Fermi surface ($q \approx q_\mathrm{F}$), before saturating to the bound imposed by the fermionic sum rule. (i) is in excellent agreement with the INS results; (ii) may be tested by future experiments, e.g., by EELS if the resolution can be further increased.

G. Comparison with $\mathbf{CeCu_{5.9}Au_{0.1}}$

INS investigations of the heavy fermion compound $CeCu_{5.9}Au_{0.1}$ represent early evidence for Kondo destruction quantum criticality (*5*). It is instructive to compare our results on $Ce_3Pd_{20}Si_6$ with those experiments. At Au concentrations x slightly above 0.1, $CeCu_{6-x}Au_x$ exhibits long-range incommensurate AFM order with the ordering wavevector $\boldsymbol{q} \approx (0.625\,0\,0.275)$ (*55*). The INS data we use for our analysis, measured at the IRIS spectrometer at ISIS, were taken at this $\boldsymbol{q}$ as well as other wavevectors on a rod-like structure that reflects the quasi-2D correlations; the magnetic response was found to be similar for these positions (*5*). We rely on the absolute values of the dynamical spin correlation function $S(\boldsymbol{q}, \omega)$ as presented in Fig. 4A of that work, including correct background and absorption corrections. The lowest energy transfer was

0.05 meV, which is more than 3 times larger than in our case (0.015 meV). We use Eqn. 4 to calculate f_{Q}. It reaches a value of 10.1 at the lowest temperature. To extract nQFI via Eqn. 5, we use $S = 1/2$ and $g = 1.5$ (*5, 56*), and $c = 2$ for isotropic scattering (*21*), resulting in $\mathrm{nQFI} = f_{\mathrm{Q}}/4.5 = 2.24$. As explained next, we do not consider this value to be reliable.

In Fig. S10, we compare $Ce_3Pd_{20}Si_6$ with $CeCu_{5.9}Au_{0.1}$, both in terms of the dynamical structure factor $S(\omega)$ measured at the lowest temperature and in terms of the QFI density $f_{\mathrm{Q}}(T)$. In $S(\omega)$ (Fig. S10A), two differences appear: (i) the rise of $S(\omega)$ towards the lowest energies is much weaker in $CeCu_{5.9}Au_{0.1}$ than in $Ce_3Pd_{20}Si_6$; (ii) the decrease of $S(\omega)$ towards high energies is much weaker in $CeCu_{5.9}Au_{0.1}$ than in $Ce_3Pd_{20}Si_6$. The former might be due to the lower energy resolution of the measurements on $CeCu_{5.9}Au_{0.1}$, the latter might be due to imperfect background subtraction (e.g. it seems that no empty sample holder measurements were done for $CeCu_{5.9}Au_{0.1}$). As f_{Q} is proportional to the integral of $S(\omega)$ (in principle up to $\omega \to \infty$), imperfect background subtraction will overestimate f_{Q}. Finite instrument resolution, by contrast, will underestimate f_{Q}.

The comparison of the temperature dependences of f_{Q} (scaled to the respective values at 4 K) of the two compounds (Fig. S10B) is also instructive. Whereas the temperature dependence is very similar above 1.5 K, the rise towards the lowest temperatures is much weaker in $CeCu_{5.9}Au_{0.1}$ than in $Ce_3Pd_{20}Si_6$. Given the differences discussed above, we conjecture that higher resolution data on $CeCu_{5.9}Au_{0.1}$ would lead to a better agreement between the two materials. Of course, experimental confirmation is needed. We can also not rule out the interesting possibility that the QFI density and thus the entanglement depth is larger in a 3D material such as $Ce_3Pd_{20}Si_6$ than in a quasi-2D material such as $CeCu_{5.9}Au_{0.1}$.

H. Measurement of $Ce_3Pd_{20}Si_6$ at 5.8 T

The same $Ce_3Pd_{20}Si_6$ sample was studied in a separate experiment at ThALES (*19*), in a magnetic field of $5.8\,\mathrm{T}\,||\,[0\,0\,1]$, i.e. distinctly away from the quantum critical point (QCP) at 1.73 T (Fig. S11), and with improved resolution and statistics as explained in Sect. D, Additional measurements with higher resolution. Data were taken at 80 mK, at the wavevector $(1\,0\,0)$, which is equivalent to $(0\,\bar{1}\,0)$ (Sect. C).

The same analysis was performed as for the data measured at the QCP (Sect. D), except for the following: (i) because the cryostat background measurement could only be done without the dilution refrigerator unit, it was less precise and had to be corrected by subtracting a Gaussian (second blue curve peaked near $-0.07\,\mathrm{meV}$); (ii) three magnon modes are present at 5.8 T, which required adding three Lorentzian functions to the fit (three green curves at finite positive energies). As in Fig. S4B, the normalized residuals (Eqn. S6) scatter statistically around zero. Their sum (Eqn. S7) is 2.00×10^{-2}, which corresponds to $\chi^2_{\mathrm{red}} = 1.07$. The QFI extracted for the central Lorentzian is $f_{\mathrm{Q}} = 1.77 \pm 0.14$, providing evidence for bipartite entanglement. That the entanglement is finite even far away from the quantum critical field may at first be surprising. However, according to the phase diagram of $Ce_3Pd_{20}Si_6$ for this field direction (Fig. 2A of (*9*)), at 5.8 T, the quantum critical fan extends down to approximately 0.5 K. Using $\hbar\omega = k_{\mathrm{B}}T$, this corresponds to about 0.04 meV, an energy slightly less than one FWHM away from the peak (at $E = 0$). The tail beyond 1 FWHM, where a Lorentzian function decays approximately as $1/x^2$, contributes almost 30% to the total integral of the Lorentzian and will, via Eqns. 1 and 3, contribute appreciably to f_{Q}. This reveals that the QFI can also detect high-energy traces of quantum criticality, even if the QCP is far away. This can be valuable in the search for new quantum critical points.

Note that we excluded the magnon modes in the determination of f_{Q}. Integrating over the three magnon modes separately gives f^i_{Q} of 0.59 ± 0.02, 0.60 ± 0.03, and 0.32 ± 0.02 for

the three modes with increasing energy, thus each representing a separable state. Including them in the integral yields $f_\mathrm{Q} = 3.3 \pm 0.2$, which, erroneously, would have suggested 4-partite entanglement. This observation shows that great care must be taken when assessing multipartite entanglement of quantum critical fluctuations. If other modes are not neatly separated, they will add to f_Q and suggest higher multipartite entanglement than actually present.

I. Why 9-partite entanglement is a conservative lower bound

Finally, we summarize why our estimate of 9-partite entanglement in $Ce_3Pd_{20}Si_6$ is conservative and we expect future experiments on either this or other strange metal compounds to demonstrate even higher multipartite entanglement:

1. We probed the material far away from the ordering wavevector of the AFQ phase suppressed at the investigated QCP. According to the EDMFT treatment of Kondo destruction quantum criticality (*1*), the dynamical spin susceptibility and thus also the dynamical structure factor and the QFI are maximal at the ordering wavevector. We deliberately chose a different wavevector to ascertain that the signal is entirely due to quantum fluctuations from Kondo destruction.

2. We converted the QFI density f_Q to the normalized QFI, nQFI, by assuming that the magnetic field has induced the maximal value of a full Bohr magneton on top of the fluctuating electric quadrupole moments. Any reduction of this moment will increase nQFI as the square of the reduction factor (Sect. E).

3. The phase boundaries of $Ce_3Pd_{20}Si_6$ depend sensitively on the direction of the magnetic field. Any misalignment of the field from the intended crystallographic direction will result in a deviation from the precise quantum critical field, where the QFI is expected to be maximal.

4. The integral of Eqn. 4 was not taken from 0 to ∞ but only in the range where data are available.
5. The spectrometer has finite resolution, smearing out data at the lowest energies.
6. The lowest accessed temperature was only one order of magnitude below the ordering temperature of the AFQ phase suppressed at the QCP. f_{Q} will likely increase further at lower temperatures.

We hope this list will guide the search for other strange metals with high multipartite entanglement.

References

1. Si, Q., Rabello, S., Ingersent, K. & Smith, J. Locally critical quantum phase transitions in strongly correlated metals. *Nature* **413**, 804 (2001).

2. Hertz, J. A. Quantum critical phenomena. *Phys. Rev. B* **14**, 1165–1184 (1976).

3. Millis, A. J. Effect of a nonzero temperature on quantum critical points in itinerant fermion systems. *Phys. Rev. B* **48**, 7183–7196 (1993).

4. Grempel, D. R. & Si, Q. Locally critical point in an anisotropic Kondo lattice. *Phys. Rev. Lett.* **91**, 026401 (2003).

5. Schröder, A., Aeppli, G., Coldea, R., Adams, M., Stockert, O., v. Löhneysen, H., Bucher, E., Ramazashvili, R. & Coleman, P. Onset of antiferromagnetism in heavy-fermion metals. *Nature* **407**, 351–355 (2000).

6. Liu, C.-C., Paschen, S. & Si, Q. Quantum criticality enabled by intertwined degrees of freedom. *Proc. Natl. Acad. Sci. U.S.A.* **120**, e2300903120 (2023).

7. Schultz, D. J., Han, S. & Kim, Y. B. Quantum impurity model for two-stage multipolar ordering and Fermi surface reconstruction. *Phys. Rev. B* **108**, L060401 (2023).

8. Custers, J., Lorenzer, K., Müller, M., Prokofiev, A., Sidorenko, A., Winkler, H., Strydom, A. M., Shimura, Y., Sakakibara, T., Yu, R., Si, Q. & Paschen, S. Destruction of the Kondo effect in the cubic heavy-fermion compound $Ce_3Pd_{20}Si_6$. *Nat. Mater.* **11**, 189 (2012).

9. Martelli, V., Cai, A., Nica, E. M., Taupin, M., Prokofiev, A., Liu, C.-C., Lai, H.-H., Yu, R., Ingersent, K., Küchler, R., Strydom, A. M., Geiger, D., Haenel, J., Larrea, J., Si, Q. & Paschen, S. Sequential localization of a complex electron fluid. *Proc. Natl. Acad. Sci. U.S.A.* **116**, 17701 (2019).

10. Matsumura, T., Yonemura, T., Kunimori, K., Sera, M., Iga, F., Nagao, T. & Igarashi, J.-i. Antiferroquadrupole order and magnetic field induced octupole in CeB_6. *Phys. Rev. B* **85**, 174417 (2012).

11. Horodecki, R., Horodecki, P., Horodecki, M. & Horodecki, K. Quantum entanglement. *Rev. Mod. Phys.* **81**, 865–942 (2009).

12. Frérot, I., Fadel, M. & Lewenstein, M. Probing quantum correlations in many-body systems: a review of scalable methods. *Rep. Prog. Phys.* **86**, 114001 (2023).

13. Tóth, G. & Apellaniz, I. Quantum metrology from a quantum information science perspective. *J. Phys. A-Math.* **47**, 424006 (2014).

14. Hauke, P., Heyl, M., Tagliacozzo, L. & Zoller, P. Measuring multipartite entanglement through dynamic susceptibilities. *Nat. Phys.* **12**, 778–782 (2016).

15. Mazza, F., Portnichenko, P. Y., Safiri, M., Bohem, M., Steffens, P., Jimenez Ruiz, M., Inosov, D. S. & Paschen, S. Institute Laue Langevin (ILL), Proposal number 4-03-1754, DOI: doi.ill.fr/10.5291/ILL-DATA.4-03-1754.

16. Mazza, F., Portnichenko, P. Y., Avdoshenko, S., Steffens, P., Boehm, M., Choi, E. S., Nikolo, M., Yan, X., Prokofiev, A., Paschen, S. & Inosov, D. S. Cascade of magnetic-field-driven quantum phase transitions in $Ce_3Pd_{20}Si_6$. *Phys. Rev. B* **105**, 174429 (2022).

17. NIST Inorganic Crystal Structure Database. NIST Standard Reference Database No. 3, National Institute of Standards and Technology, Gaithersburg MD, 20899, DOI:10.18434/M32147 (retrieved 2024-02-21).

18. Portnichenko, P. Y., Nikitin, S. E., Prokofiev, A., Paschen, S., Mignot, J.-M., Ollivier, J., Podlesnyak, A., Meng, S., Lu, Z. & Inosov, D. S. Evolution of the propagation vector of

antiferroquadrupolar phases in $Ce_3Pd_{20}Si_6$ under magnetic field. *Phys. Rev. B* **99**, 214431 (2019).

19. Mazza, F., Naessens, M., Forslund, O. K., Yan, X., Steffens, P., Prokofiev, A. & Paschen, S. Institute Laue Langevin (ILL), Institute Laue Langevin (ILL), Proposal number 4-03-1776, DOI: doi.ill.fr/10.5291/ILL-DATA.4-03-1776.

20. Chakrabarti, B., Pezzoli, M. E., Sordi, G., Haule, K. & Kotliar, G. α-γ transition in cerium: Magnetic form factor and dynamic magnetic susceptibility in dynamical mean-field theory. *Phys. Rev. B* **89**, 125113 (2014).

21. Xu, G., Xu, Z. & Tranquada, J. M. Absolute cross-section normalization of magnetic neutron scattering data. *Rev. Sci. Instrum.* **84**, 083906 (2013).

22. Portnichenko, P. Y., Cameron, A. S., Surmach, M. A., Deen, P. P., Paschen, S., Prokofiev, A., Mignot, J.-M., Strydom, A. M., Telling, M. T. F., Podlesnyak, A. & Inosov, D. S. Momentum-space structure of quasielastic spin fluctuations in $Ce_3Pd_{20}Si_6$. *Phys. Rev. B* **91**, 094412 (2015).

23. Stassis, C., Loong, C., Kline, G. R., McMasters, O. D. & Gschneidner, J., K. A. Field induced magnetic form factor of γ-Ce. *J. Appl. Phys.* **49**, 2113–2114 (1978).

24. Murani, A. P., Levett, S. J. & Taylor, J. W. Magnetic form factor of α-Ce: Towards understanding the magnetism of cerium. *Phys. Rev. Lett.* **95**, 256403 (2005).

25. Furrer, A., Mesot, J. & Strässle, T. *Neutron Scattering in Condensed Matter Physics* (World Scientific, Singapore, 2009), 1 edn.

26. Scheie, A., Laurell, P., Samarakoon, A. M., Lake, B., Nagler, S. E., Granroth, G. E., Okamoto, S., Alvarez, G. & Tennant, D. A. *Phys. Rev. B* **103**, 224434 (2021) and erratum: *Phys. Rev. B* **107**, 059902 (2023).

27. Laurell, P., Scheie, A., Mukherjee, C. J., Koza, M. M., Enderle, M., Tylczynski, Z., Okamoto, S., Coldea, R., Tennant, D. A. & Alvarez, G. *Phys. Rev. Lett.* **127**, 037201 (2021) and erratum: *Phys. Rev. Lett.* **130**, 129902 (2023).

28. Menon, V., Sherman, N. E., Dupont, M., Scheie, A. O., Tennant, D. A. & Moore, J. E. Multipartite entanglement in the one-dimensional spin-$\frac{1}{2}$ Heisenberg antiferromagnet. *Phys. Rev. B* **107**, 054422 (2023).

29. Scheie, A. O., Ghioldi, J., E. A. Xing, Paddison, J. A. M., Sherman, N. E., Dupont, M., Sanjeewa, L. D., Lee, S., Woods, A. J., Abernathy, D., Pajerowski, D. M., Williams, T. J., Zhang, S.-S., Manuel, L. O., Trumper, A. E., Pemmaraju, C. D., Sefat, A. S., Parker, D. S., Devereaux, T. P., Movshovich, R., Moore, J. E., Batista, C. D. & Tennant, D. A. Proximate spin liquid and fractionalization in the triangular antiferromagnet $KYbSe_2$. *Nat. Phys.* **20**, 74–81 (2024).

30. Fang, Y., Mahankali, M., Wang, Y., Chen, L., Hu, H., Paschen, S. & Si, Q. Amplified multipartite entanglement witnessed in a quantum critical metal. *Nat. Commun.* **16**, 2498 (2025).

31. Johnston, D. F. On the theory of the electron orbital contribution to the scattering of neutrons by magnetic ions in crystals. *Proc. Phys. Soc.* **88**, 37 (1966).

32. Portnichenko, P. Y., Paschen, S., Prokofiev, A., Vojta, M., Cameron, A. S., Mignot, J.-M., Ivanov, A. & Inosov, D. S. Incommensurate short-range multipolar order parameter of phase II in $Ce_3Pd_{20}Si_6$. *Phys. Rev. B* **94**, 245132 (2016).

33. Landau, L. D. & Lifshitz, E. M. *Statistical Physics, Part 1* (Pergamon Press, Oxford, 1980).

34. Santini, P., Carretta, S., Amoretti, G., Caciuffo, R., Magnani, N. & Lander, G. H. Multipolar interactions in f-electron systems: The paradigm of actinide dioxides. *Rev. Mod. Phys.* **81**, 807–863 (2009).

35. Lee, S., Trebst, S., Kim, Y. B. & Paramekanti, A. Landau theory of multipolar orders in $\mathrm{Pr}(Y)_2X_{20}$ Kondo materials ($Y = \mathrm{Ti}$, V, Rh, Ir; $X = \mathrm{Al}$, Zn). *Phys. Rev. B* **98**, 134447 (2018).

36. Patri, A. S., Sakai, A., Lee, S., Paramekanti, A., Nakatsuji, S. & Kim, Y. B. Unveiling hidden multipolar orders with magnetostriction. *Nat. Commun.* **10**, 4092 (2019).

37. Shiina, R., Shiba, H. & Thalmeier, P. Magnetic-field effects on quadrupolar ordering in a Γ_8-quartet system CeB_6. *J. Phys. Soc. Jpn.* **66**, 1741 (1997).

38. Thalmeier, P., Shiina, R., Shiba, H., Takahashi, A. & Sakai, O. Temperature and field dependence of multipolar excitations in CeB_6. *J. Phys. Soc. Jpn.* **72**, 3219–3225 (2003).

39. Takigawa, M., Yasuoka, H., Tanaka, T. & Ishizawa, Y. NMR study on the spin structure of CeB_6. *J. Phys. Soc. Jpn.* **52**, 728 (1983).

40. Rossat-Mignot, J. *Methods of Experimental Physics, Vol. 23C, p. 69* (Academic Press, New York, 1987).

41. Tsuji, S., Sera, M. & Kojima, K. Analysis of ^{11}B-NMR in phase II of CeB_6. *J. Phys. Soc. Jpn.* **70**, 41–44 (2001).

42. Sakakibara, T., Tayama, T., Onimaru, T., Aoki, D., Onuki, Y., Sugawara, H., Aoki, Y. & Sato, H. Quadrupole ordering and multipole interactions in Pr-based compounds. *J. Phys.: Condens. Matter* **15**, S2055 (2003).

43. Sato, T. J., Ibuka, S., Nambu, Y., Yamazaki, T., Hong, T., Sakai, A. & Nakatsuji, S. Ferro-quadrupolar ordering in $PrTi_2Al_{20}$. *Phys. Rev. B* **86**, 184419 (2012).

44. Thalmeier, P., Akbari, A. & Shiina, R. *Rare-Earth Borides*, chap. 8, Multipolar order and excitations in rare-earth boride Kondo systems, 615–690 (ed. D. S. Inosov, Jenny Stanford Publishing, 2021).

45. Assaad, F. F., Bercx, M., Goth, F., Götz, A., Hofmann, J. S., Huffman, E., Liu, Z., Toldin, F. P., Portela, J. S. E. & Schwab, J. The ALF (Algorithms for Lattice Fermions) project release 2.4. Documentation for the auxiliary-field quantum Monte Carlo code. *SciPost Phys. Codebases* 1–v2.4 (2025).

46. Blankenbecler, R., Scalapino, D. J. & Sugar, R. L. Monte Carlo calculations of coupled boson-fermion systems. I. *Phys. Rev. D* **24**, 2278–2286 (1981).

47. White, S. R., Scalapino, D. J., Sugar, R. L., Loh, E. Y., Gubernatis, J. E. & Scalettar, R. T. Numerical study of the two-dimensional Hubbard model. *Phys. Rev. B* **40**, 506–516 (1989).

48. Sorella, S., Baroni, S., Car, R. & Parrinello, M. A novel technique for the simulation of interacting fermion systems. *Europhys. Lett.* **8**, 663 (1989).

49. Assaad, F. F. & Evertz, H. G. *World-line and Determinantal Quantum Monte Carlo Methods for Spins, Phonons and Electrons*, 277–356 (Springer Berlin Heidelberg, Berlin, Heidelberg, 2008).

50. Beach, K. S. D. Identifying the maximum entropy method as a special limit of stochastic analytic continuation, *arXiv:cond-mat/0403055* (2004).

51. Sandvik, A. W. Stochastic method for analytic continuation of quantum Monte Carlo data. *Phys. Rev. B* **57**, 10287–10290 (1998).

52. Shao, H. & Sandvik, A. W. Progress on stochastic analytic continuation of quantum Monte Carlo data. *Phys. Rep.* **1003**, 1–88 (2023).

53. Danu, B., Liu, Z., Assaad, F. F. & Raczkowski, M. Zooming in on heavy fermions in Kondo lattice models. *Phys. Rev. B* **104**, 155128 (2021).

54. Malla, R. K., Weichselbaum, A., Wei, T.-C. & Konik, R. M. Detecting multipartite entanglement patterns using single particle Green's functions, *arXiv:2310.05870* (2023).

55. v. Löhneysen, H., Neubert, A., Pietrus, T., Schröder, A., Stockert, O., Tutsch, U., Loewenhaupt, M., Rosch, A. & Wölfle, P. Magnetic order and transport in the heavy-fermion system $CeCu_{6-x}Au_x$. *Eur. Phys. J. B* **5**, 447–455 (1998).

56. Ōnuki, Y., Shimizu, Y. & Komatsubara, T. Anisotropic magnetic property of Kondo lattice substance: $CeCu_6$. *J. Phys. Soc. Jpn.* **54**, 304–311 (1985).

Supplementary Tables

Table S1: **Resolution volume determined via incoherent elastic scattering.** $\overline{G}$ is the amplitude of Gaussian fits to the incoherent elastic scattering intensity from $Ce_3Pd_{20}Si_6$ at $\boldsymbol{q} = (0\,\bar{1}\,0)$, averaged over all temperatures ($\leq\ 10\,\mathrm{K}$), $\overline{I_{\mathrm{inc}}^{\mathrm{int}}}(0\,\bar{1}\,0)$ the mean integrated intensity of these Gaussian fits, and $\frac{d\sigma}{d\Omega}|_{\mathrm{inc}}^{\mathrm{el}}$ the cross-section for incoherent elastic scattering from one unit cell of $Ce_3Pd_{20}Si_6$, as determined from (*17*). Details on the evaluation of the values and error bars are given in Sect. D.

$\overline{G}$ (-)	$\overline{I_{\mathrm{inc}}^{\mathrm{int}}}(0\,\bar{1}\,0)$ (meV)	$\frac{d\sigma}{d\Omega}\|_{\mathrm{inc}}^{\mathrm{el}}$ (b)	R_0 (meV/b)
9613 ± 889	846 ± 78	0.601 ± 0.017	1409 ± 137

Table S2: **Alternative estimates of the incoherent elastic scattering contribution.** $I_{\mathrm{inc}}^{\mathrm{int}}$ are the integrated intensities of Gaussian fits to incoherent elastic scattering from the $Ce_3Pd_{20}Si_6$ sample in the cryostat at low temperatures (temperature average from Table S1), from a vanadium standard sample in the cryostat at room temperature, and the $Ce_3Pd_{20}Si_6$ sample outside the cryostat at room temperature. Details on the evaluation of the values and error bars are given in Sect. D. All results agree within error bars, but the relative errors are smallest for our analysis (top line).

$I_{\mathrm{inc}}^{\mathrm{int}}$ (meV)	f_{Q}	T (K)	comment
846 ± 78	8.2 ± 0.9	$0.06 - 10$	our analysis (Table S1)
866 ± 119	7.7 ± 1.0	300	vanadium in cryostat
861 ± 133	7.8 ± 1.2	300	sample outside cryostat

Supplementary Figures

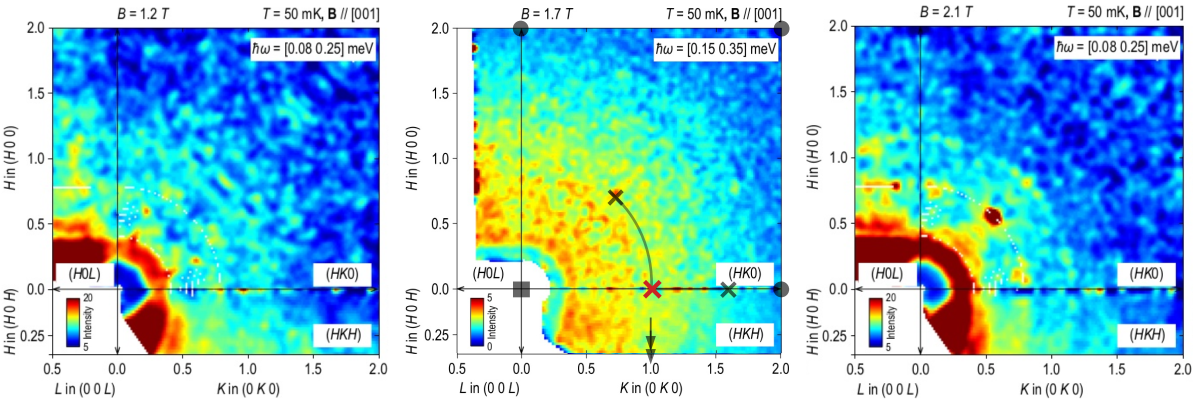

Figure S1: **Evolution of INS intensity of $Ce_3Pd_{20}Si_6$ with field.** Constant-energy maps at 1.2 T (left), 1.7 T (center, from Fig. 1B), and 2.1 T (right). The energy integration ranges of the time-of-flight data are indicated. In orthogonal momentum directions with respect to each plane, integration was done within ± 0.08 r.l.u. for the center panel, and ± 0.1 r.l.u. for the others. A broad momentum space distribution of low-energy intensity is seen only at 1.7 T, i.e. close to the Kondo destruction QCP at 1.73 T, confirming that this intensity arises from quantum critical fluctuations of Kondo destruction type. Adapted from (*18*).

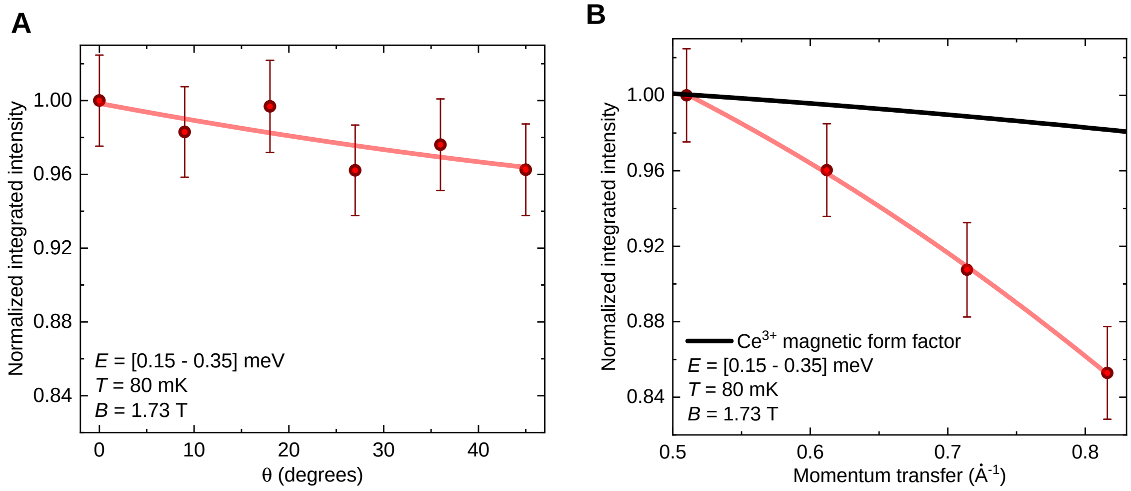

Figure S2: **Wavevector dependence of the energy-integrated intensity.** Data taken on the triple-axis spectrometer ThALES (ILL, Grenoble) at the quantum critical field of 1.73 T and at 80 mK, integrated from 0.15 meV to 0.35 meV (as in Fig. 1B of the main text), for two different trajectories: (**A**) On a ring of constant $|\boldsymbol{q}|$, with the angles $\theta = 0°$ and $45°$ corresponding to (1 0 0) and ($\frac{1}{\sqrt{2}}$ $\frac{1}{\sqrt{2}}$ 0), respectively. (**B**) Along the k_y direction, from (0 1 0) to (0 1.6 0). The momentum dependence of the magnetic form factor of Ce (*20*) is shown for comparison. Note that the data in (A) and (B) contain (empty cryostat and incoherent scattering) background contributions; nevertheless, the observed trends are significant and reveal that the quantum critical signal has a distinct momentum space structure. The error bars represent statistical uncertainties of the measured intensity ($\Delta I = \sqrt{I}$) and of the integrated spectral weight according to Simpson's rule (see Supplementary Information Sect. D).

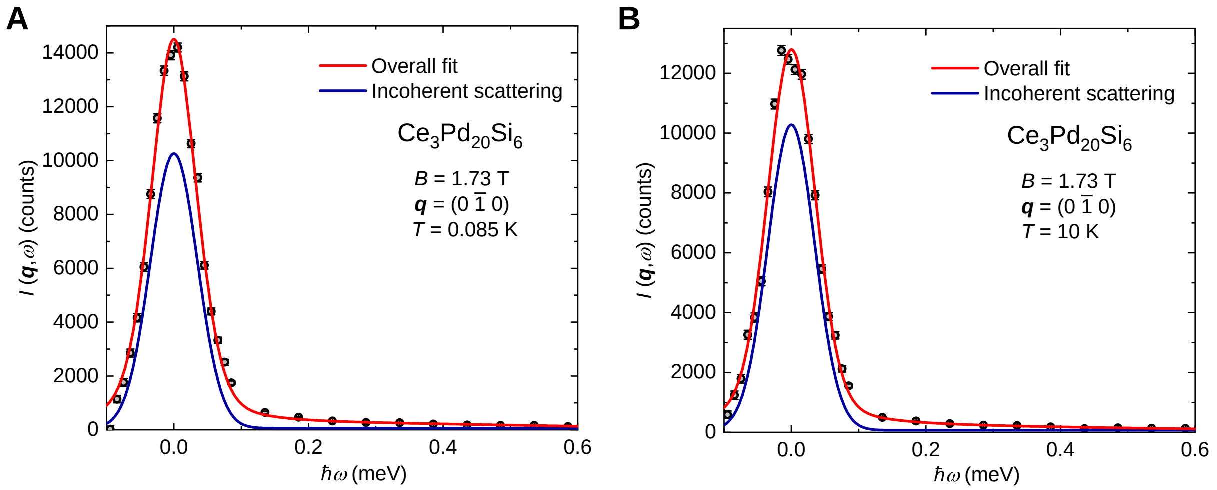

Figure S3: **Determination of the incoherent elastic background from the sample.** Displayed is the neutron scattering intensity I of $Ce_3Pd_{20}Si_6$ at $\boldsymbol{q} = (0\,\bar{1}\,0)$, with a magnetic field of 1.73 T along $[0\,0\,1]$ measured at 0.085 K (**A**) and 10 K (**B**). The overall fit (red) contains a Gaussian (blue) that represents the incoherent elastic background from the sample. The error bars represent statistical propagated errors and uncertainties after empty cryostat background subtraction (see Supplementary Information Sect. D).

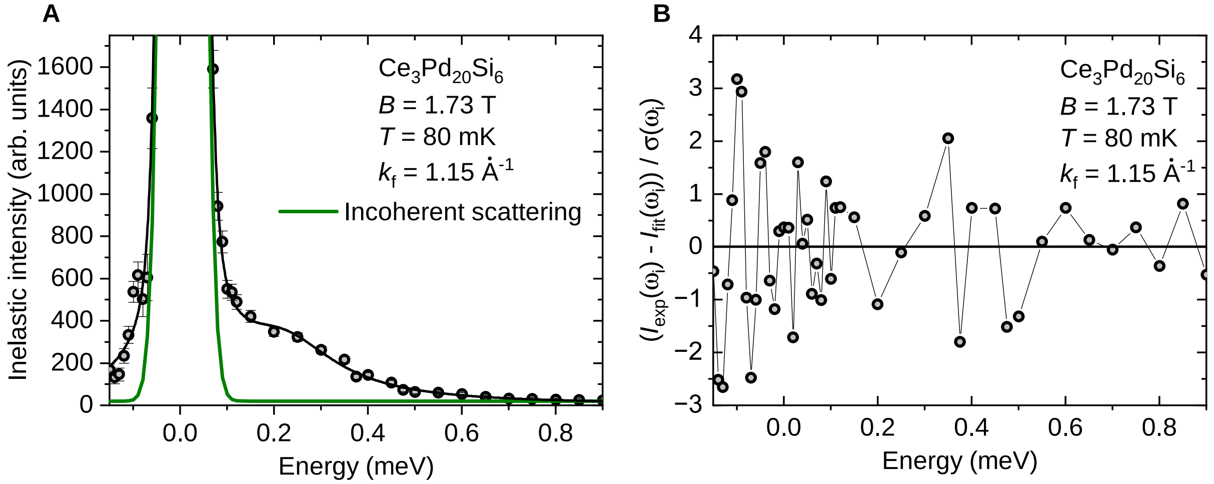

Figure S4: **Higher-resolution INS measurement of $\mathbf{Ce_3Pd_{20}Si_6}$.** (**A**) Background-subtracted and absorption-corrected total INS intensity of $Ce_3Pd_{20}Si_6$ (symbols), containing coherent and incoherent contributions. The data were taken at $\boldsymbol{q} = (1\,0\,0)$, $T = 80\,\mathrm{mK}$, and the critical magnetic field of 1.73 T applied along $[0\,0\,1]$, at ThALES with improved instrumental resolution (compared to the data in the main part) using the final wavevector $k_\mathrm{f} = 1.15\,\mathrm{\AA}^{-1}$ and better statistics (*19*). The fit (black line) composed of a Gaussian and two Lorentzians, describes the data very well. The incoherent contribution is also shown separately as a green line. (**B**) The residuals (data minus fit) fluctuate statistically around zero, without a systematic structure and a reduced chi-squared value of $\chi^2_\mathrm{red} = 2.16$, indicating that the used model captures the data very well, with deviations mostly due to statistical noise and experimental uncertainty. The error bars represent statistical propagated errors and uncertainties after empty cryostat background subtraction (see Supplementary Information Sect. D).

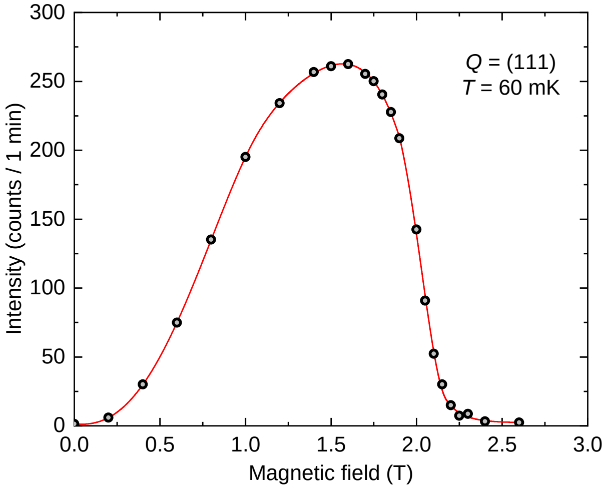

Figure S5: **Magnetic intensity of antiferroquadrupolar phase II of $Ce_3Pd_{20}Si_6$.** Magnetic-field dependence of the magnetic intensity associated with the antiferroquadrupolar (AFQ) phase II of $Ce_3Pd_{20}Si_6$, measured at 60 mK at its ordering wavevector $(1\,1\,1)$, for magnetic fields (nominally) applied along $[0\,0\,1]$ (that the critical field of about 2.4 T is distinctly larger than in the present experiments indicates that there was some misalignment; for fields along $[1\,\bar{1}\,0]$, for instance, phase II extends to beyond 9 T). Note that even though phase II is known to set in below about 0.4 K in zero field, the magnetic Bragg intensity is zero in zero field and builds up smoothly with increasing field before being suppressed at the quantum critical field. This provides evidence that the applied magnetic field induces dipole moments on top of the primary quadrupole moments, which make the primary order visible to the neutrons. Adapted from Fig. 5e of (*32*).

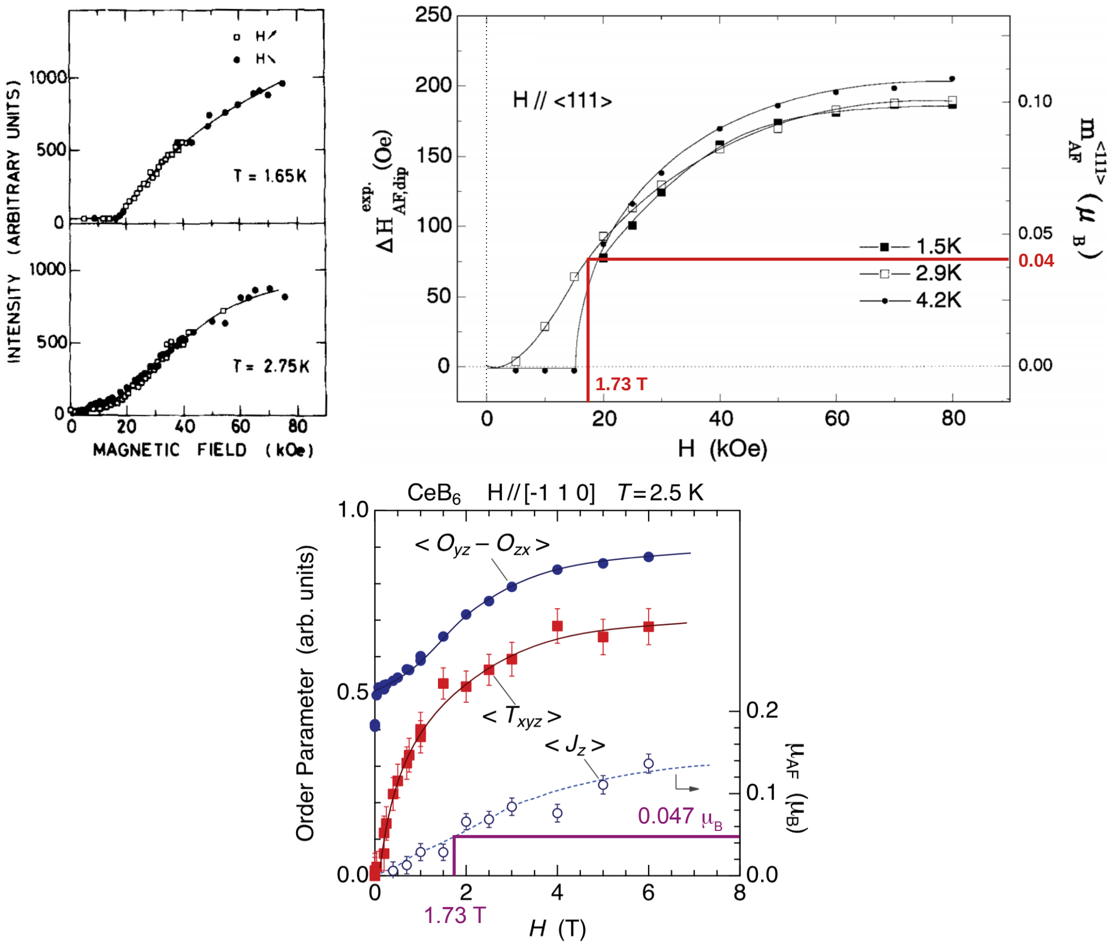

Figure S6: **Magnetic field effect on the AFQ phase in CeB_6.** (left) Magnetic field induced Bragg intensity at the AFQ ordering wavevector $(\frac{1}{2}\,\frac{1}{2}\,\frac{1}{2})$ for field along $(0\,1\,1)$ (*40*). Figure from (*40*). (right) Magnetic field induced dipole moment at the Ce site in the AFQ phase in CeB_6, as determined in (*39, 41*) from the splitting of the ^{11}B NMR line when cooling CeB_6 from the paramagnetic phase I into the AFQ phase II. Plotted is the moment for fields along the $(1\,1\,1)$ direction. Figure adapted from (*41*). (bottom) Compilation of data at 2.5 K from the same experiments (*39–41*) for field along $(\bar{1}\,1\,0)$, with the dashed line showing the field dependence of the dipole moment measured by neutron diffraction (*40*) with the absolute value deduced from NMR (*39,41*). Adapted from (*10*). For the $(0\,0\,1)$ direction of relevance to us, the moment is 20% smaller than for fields along $(1\,1\,1)$, thus about $0.033\,\mu_B$. A further correction by the rate of increase of the AFQ phase boundary with field contributes a further reduction factor of 4, i.e., to an estimated moment of below $0.01\,\mu_B$ at 1.73 T in $Ce_3Pd_{20}Si_6$, as detailed in Sect. E.

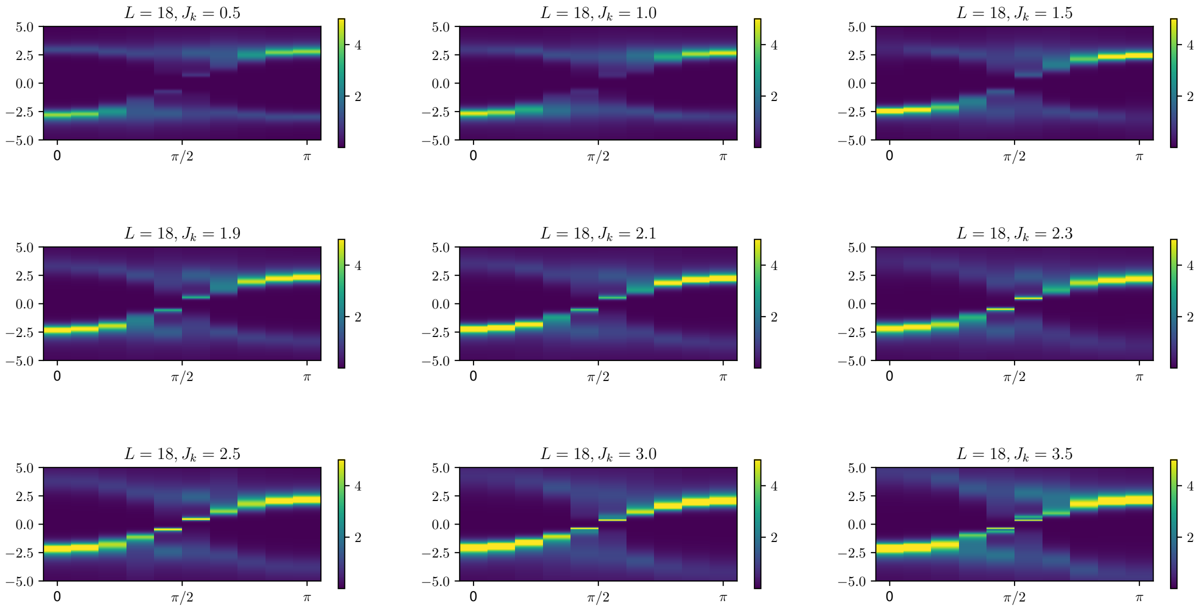

Figure S7: **Composite fermion spectral function across the Kondo destruction transition.** The colour scale shows the fermion spectral function $A(q, \omega)$, which is investigated across the Kondo destruction transition by scanning the Kondo coupling J_{K}. The x-axis of all plots shows the wavevector q while the y-axis shows the frequency ω. The data clearly shows the appearance of a composite fermion quasiparticle across the Kondo destruction transition at $J_{\mathrm{K}}{\approx}2$.

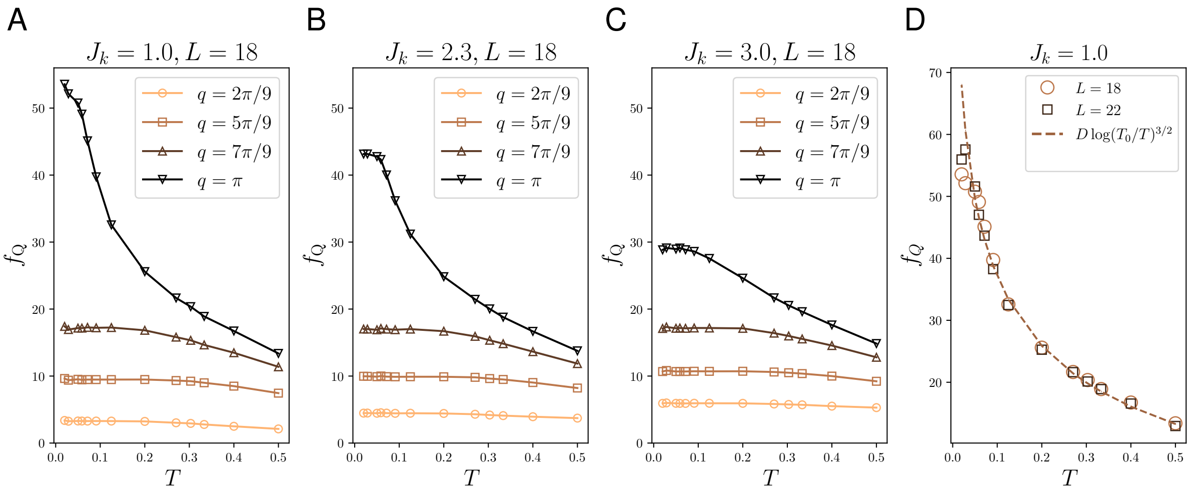

Figure S8: **QFI density for spins across the Kondo destruction transition.** In the Kondo destruction phase ($J_{\rm K} = 1$, **A**), the QFI density at the wavevector $q = \pi$ of critical AFM fluctuations grows with decreasing temperature, and is consistent with the $\log^{3/2}(1/T)$ form of the isolated spin 1/2 chain (*28*) (**D**). In the Kondo-screened phase ($J_{\rm K} = 2.3$ and 3.9, **B** and **C**), it saturates at low temperatures for all wavevectors. Error bars (smaller than the symbol sizes) result from statistical errors of the ALF-library (*45*) implementation of stochastic maximum entropy calculations (*50–52*) used to obtain the QFI from imaginary-time data obtained in QMC simulations.

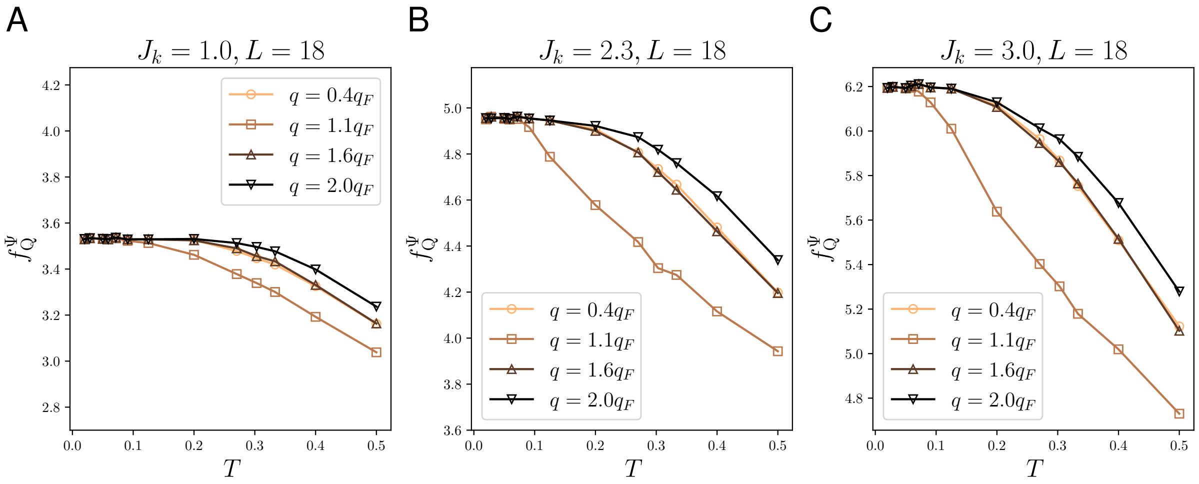

Figure S9: **Composite fermion QFI density across the Kondo destruction transition.** In the Kondo destruction phase ($J_{\mathrm{K}} = 1$, **A**), the QFI density has a weak temperature dependence for all wavevectors, whereas for the Kondo-screened phase ($J_{\mathrm{K}} = 2.3$ and 3, **B** and **C**), we see a pronounced temperature dependence for wavevectors near the Fermi wavevector $q_{\mathrm{F}} = \pi/2$ ($q = 1.1q_{\mathrm{F}}$ curves). As mentioned in Sect. F, $f_{\mathrm{Q}}^{\Psi}(T = 0) = -\frac{4}{N}\sum_i \left\langle \vec{S}_i \cdot \vec{c}_i^{\dagger}\vec{\sigma}\vec{c}_i \right\rangle + 4S(S+1)$. Thus, the local spin-spin correlations between the spin and the conduction electrons determine the momentum-independent value of $f_{\mathrm{Q}}^{\Psi}(T = 0)$. At $J_{\mathrm{K}} = 3.0$ (C) we observe a well-defined quasiparticle in the vicinity of the Fermi energy, $q = 1.1q_{\mathrm{F}}$, such that the approach to a saturation value is quicker than a linear-in-T law. Note that for a Fermi liquid in the thermodynamic limit, we would expect a T^2 law at the Fermi energy. In contrast, in the Kondo breakdown phase at $J_k = 1.0$ (A), where the quasiparticle pole gives way to an incoherent spectrum, we observe a much weaker temperature dependence towards the saturation value. In this case, and as mentioned in Sect. F, we expect a linear-in-T behavior. Error bars (smaller than the symbol sizes) result from statistical errors of the ALF-library (*45*) implementation of stochastic maximum entropy calculations (*50–52*) used to obtain the QFI from imaginary-time data obtained in QMC simulations.

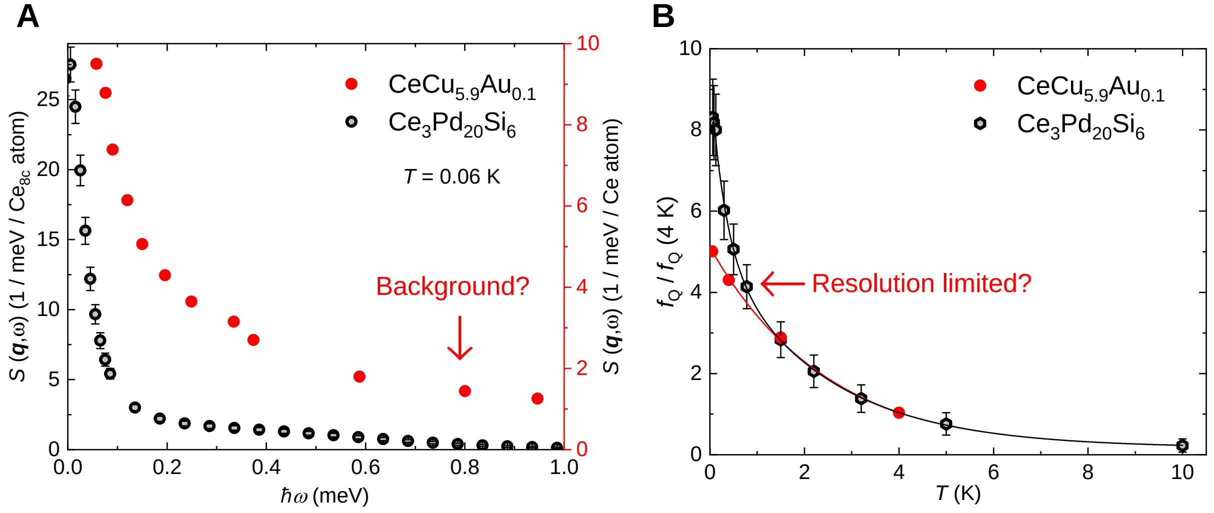

Figure S10: **Comparison of $CeCu_{5.9}Au_{0.1}$ and $Ce_3Pd_{20}Si_6$.** **(A)** Dynamical structure factor $S(\boldsymbol{q},\omega)$, measured at $\boldsymbol{q} = (0\,\bar{1}\,0)$ for $Ce_3Pd_{20}Si_6$ (left axis) and at $\boldsymbol{q} = (0.8\,0\,0)$ for $CeCu_{5.9}Au_{0.1}$ (right axis), both at the lowest accessed temperature. **(B)** Quantum Fisher information density for both compounds, normalized by the respective data point at 4 K. The data for $CeCu_{5.9}Au_{0.1}$ are taken from (*5*). The error bars for the $Ce_3Pd_{20}Si_6$ data result from statistical errors and uncertainties in other quantities via error propagation (see Supplementary Information Sect. D).

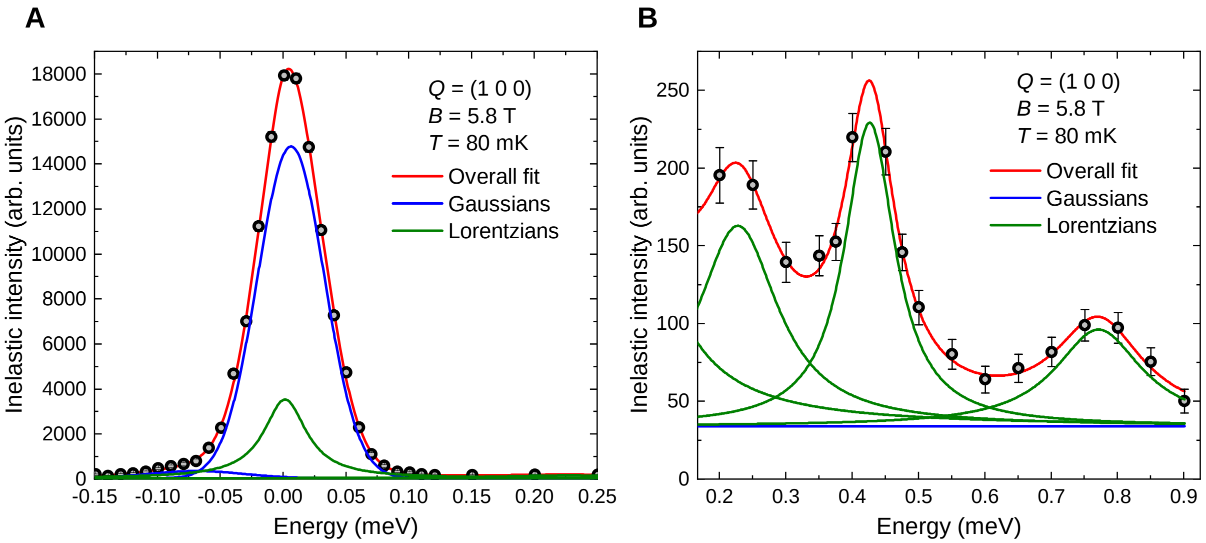

Figure S11: **Additional INS data on $Ce_3Pd_{20}Si_6$ at 5.8 T.** Energy scan at $(1\,0\,0)$, taken at 80 mK, with **(A)** displaying the low energy region and **(B)** a zoom into the higher energy region. The overall fit (red line) describes the data very well. It consists of several contributions. The two blue lines are Gaussian fits peaked at $E = 0$ and near -0.07 meV, representing the incoherent elastic signal and a residual cryostat contribution, respectively. The latter contains a constant offset. The four green lines are Lorentzian functions representing three magnon modes at finite energies, and the low-energy signal of interest to us here. The error bars represent statistical propagated errors and uncertainties after empty cryostat background subtraction (see Supplementary Information Sect. D).
